# Supplementary material for: Mott insulators with boundary zeros
Source: Nat Commun. 2023 Nov 20;14:7531. doi: 10.1038/s41467-023-42773-7 (PMC10662449; doi:10.1038/s41467-023-42773-7)
Supplement: Supplementary file 1 — Supplementary Information [file 41467_2023_42773_MOESM1_ESM.pdf]

Supplementary Information: Mott insulators with boundary zeros

CONTENTS

Supplementary Note 1. Analytic formula for the self-energy 2
- A. Derivation of the expansion 2
- B. Application to the Hubbard-model 3
- C. Correlation values 5
  - 1. Expansion about the atomic limit 5
  - 2. Numerical results 6
- D. Analytic estimation of higher-order contributions 7
- E. Topological invariants from the zeros 9
- F. Numerical topological invariant 9
- G. Application of the analytic self-energy formula to the Hubbard dimer 11
  - 1. Exact solution 11
  - 2. Analytic formula 11
  - 3. Explicit high-frequency expansion 11
  - 4. Hubbard dimer as interface 12

Supplementary Note 2. Slab calculations using the analytic formula 13

Supplementary Note 3. Additional results 14
- A. QMC vs ED for the SSH model 14
- B. Comparison of analytic formula and numerical results for an interface between SSH chains 15
- C. SSH model with broken chiral symmetry 16
- D. Appearance of zeros in the SSH model 18
- E. Numerical estimation of higher order corrections 19
- F. Tunneling through coupled region 20
- G. Dependence of the pole-zero annihilation on the slope of the edge states 22
- H. Minimal model for edge annihilation 23
- I. Orbital and spin character of the zeros 26
- J. Comparison of analytic formula and numerical results for a bulk BHZ model 28
- K. Comparison of analytic formula and numerical results for a bulk Weyl semimetal model 30
- L. Spin gap in the SSH model 32

Supplementary References 34

This Supplementary Information is structured as follows: The first section (Supplementary Note 1) describes the derivation of Eq.(1) of the main text, i.e. $\Sigma(k,\omega) = \frac{U^2/4}{\omega+\widetilde{H}_0(k)}$ and its immediate consequences. The second section (Supplementary Note 2) gives further details about the numerical calculations for the 2D TI slab calculations. Additional results and comparisons between the analytic self-energy formula and numerical calculations are shown in the third section (Supplementary Note 3).

Supplementary Note 1. ANALYTIC FORMULA FOR THE SELF-ENERGY

A. Derivation of the expansion

Our approach is based on a moments (or continued fraction) expansion of the Green's function [1–6]. Following references [7–9], the Matsubara Green's function $G_{\eta\rho}(i\omega_n, k)$ (where η and ρ describe the orbital and spin degrees of freedom, ω_n is the Matsubara frequency and k is crystal momentum) can be expressed using the Fourier transform of $G(\tau, k)$:

$$G_{\eta\rho}(i\omega_n, k) = \int_0^\beta e^{i\omega_n\tau} G_{\eta\rho}(\tau, k) d\tau. \tag{1}$$

Here τ is the imaginary time and β is the inverse temperature. This expression can be rewritten as

$$G_{\eta\rho}(i\omega_n, k) = \frac{1}{i\omega_n}\int_0^\beta (\partial_\tau e^{i\omega_n\tau}) G_{\eta\rho}(\tau, k) d\tau. \tag{2}$$

Now, integration by parts yields

$$G_{\eta\rho}(i\omega_n, k) = \frac{1}{i\omega_n}\left(-G_{\eta\rho}(\beta^-, k) - G_{\eta\rho}(0^+, k) - \int_0^\beta e^{i\omega_n\tau}(\partial_\tau G_{\eta\rho}(\tau, k))\right) d\tau. \tag{3}$$

This procedure can be repeated for the second term leading to

$$\begin{aligned} G_{\eta\rho}(i\omega_n, k) = &-\frac{1}{i\omega_n}\left(G_{\eta\rho}(\beta^-, k) + G_{\eta\rho}(0^+, k)\right) + \frac{1}{(i\omega_n)^2}\left(\partial_\tau G_{\eta\rho}(\beta, 0) + \partial_\tau G_{\eta\rho}(0, k)\right) \\ &+ \frac{1}{(i\omega_n)^3}\int_0^\beta (\partial_\tau e^{i\omega_n\tau})(\partial_\tau^2 G_{\eta\rho}(\tau, k)). \end{aligned} \tag{4}$$

Carrying out this procedure repeatedly we arrive at

$$G_{\eta\rho}(i\omega_n, k) = \frac{1}{i\omega_n}\sum_{j=0}^{\infty}(-1)^{j+1}\frac{\left(\partial_\tau^j G_{\eta\rho}(\beta^-, k) + \partial_\tau^j G_{\eta\rho}(0^+, k)\right)}{(i\omega_n)^j} = \frac{1}{i\omega_n}\sum_{j=0}^{\infty}\frac{(-1)^{j+1}C_j^{\eta\rho}}{(i\omega_n)^j}. \tag{5}$$

The derivatives of $G_{\eta\rho}(\tau, k)$ can be computed using

$$G_{\eta\rho}(\tau, k) = -\left\langle c_\eta(k,\tau)c_\rho^\dagger(k, 0)\right\rangle = -\frac{\mathrm{Tr}(e^{-\beta H}e^{H\tau}c_\eta(k, 0)e^{-H\tau}c_\rho^\dagger(k, 0))}{\mathrm{Tr}(e^{-\beta H})}. \tag{6}$$

We get

$$C_0^{\eta\rho} = (G_{\eta\rho}(0^+, k) + G_{\eta\rho}(\beta^-, k)) = -1 \tag{7}$$

$$C_1^{\eta\rho} = (\partial_\tau G(0^+, k) + \partial_\tau G(\beta^-, k)) = -\left\langle\left\{[H, c_\eta], c_\rho^\dagger\right\}\right\rangle \tag{8}$$

and in general

$$C_i^{\eta\rho} = -\left\langle\left\{\underbrace{[H, \ldots [H, c_\eta(k)]]}_{\text{i commutators}}, c_\rho^\dagger(k)\right\}\right\rangle. \tag{9}$$

The Green's function is given by

$$G(i\omega_n, k) = (i\omega_n - \varepsilon(k) - \Sigma(i\omega_n, k))^{-1} \tag{10}$$

where, depending on the degrees of freedom, all quantities can be matrices. Inverting the previous formula yields the self-energy

$$\Sigma(i\omega_n, k) = i\omega_n - \varepsilon(k) - \frac{1}{G(i\omega_n, k)} = i\omega_n - \varepsilon(k) - \frac{i\omega_n}{1 - \sum_{j=1}^{\infty} (-1)^j \frac{C_j}{(i\omega_n)^j}}. \tag{11}$$

In the limit of $i\omega_n \to \infty$ the last term can be seen as the sum of a geometric series. Therefore:

$$\begin{aligned} \Sigma(i\omega_n, k) &= i\omega_n - \varepsilon(k) - (i\omega_n) \left(1 + \sum_{n=1}^{\infty} (-1)^n \frac{C_n}{(i\omega_n)^n} + \left(\sum_{n=1}^{\infty} (-1)^n \frac{C_n}{(i\omega_n)^n} \right)^2 + \dots \right) \\ &= -\varepsilon(k) - (i\omega_n) \left(\sum_{n=1}^{\infty} (-1)^n \frac{C_n}{(i\omega_n)^n} + \left(\sum_{n=1}^{\infty} (-1)^n \frac{C_n}{(i\omega_n)^n} \right)^2 + \dots \right). \end{aligned} \tag{12}$$

Expanding in orders of $\frac{1}{\omega}$ we arrive at the following expression for the self-energy:

$$\begin{aligned} \Sigma(i\omega, k) = &-\varepsilon(k) + \mu + C_1(k) - \frac{C_2(k) + C_1^2(k)}{i\omega} \\ &+ \frac{C_3(k) + C_1(k)C_2(k) + C_2(k)C_1(k) + C_1^3(k)}{i\omega^2} + \mathcal{O}\left(\left(\frac{1}{i\omega} \right)^3 \right). \end{aligned} \tag{13}$$

Note that C_i are in general matrices. We can identify the poles of the self-energy by comparing with the expansion

$$\frac{A}{i\omega - B} = \frac{A}{i\omega} + \frac{AB}{i\omega^2} + \mathcal{O}\left(\left(\frac{1}{i\omega} \right)^3 \right). \tag{14}$$

For the deep Mott limit corrections from higher orders in the continued fraction are suppressed (see Sec.Supplementary Note 1 D) thus the first order of the high-frequency expansion is sufficient to determine the self-energy. For the Hubbard dimer discussed in section Supplementary Note 1 G we have checked that the expansions of Eq. 13 and Eq.14 coincide up to fourth order in $\frac{1}{i\omega}$.

B. Application to the Hubbard-model

In order to arrive at Eq.(1) of the main text we have to compute the commutators in Eq. (9) for the Hubbard model.

We consider a multi-orbital case with local Hubbard repulsion of strength U:

$$H = \sum_{kab\sigma} (\varepsilon_k^{ab\sigma\sigma'} - \mu \delta_{ab} \delta_{\sigma\sigma'}) c^\dagger_{ka\sigma} c_{kb\sigma'} + \frac{U}{2} \sum_{ia\sigma} n_{ia\sigma} n_{ia-\sigma} \tag{15}$$

where $\varepsilon_k^{ab\sigma\sigma'}$ is the dispersion as a function of momentum k with orbital indices a and b as well as spin degrees of freedom σ and σ'. The chemical potential is given by μ, i indicates a lattice site and n is the number operator. We call the on-site, k-independent terms of the dispersion $\varepsilon^{ab\sigma\sigma'}_{\text{on-site}} \delta_{ab}$.
Up to second order in $\frac{1}{\omega}$ (ω being either $i\omega_n$ or $\omega + i\delta$), equation (13) leads to

$$\Sigma^{ab\sigma\sigma'}(k, \omega) = \frac{U}{2} \delta_{ab} \delta_{\sigma\sigma'} + \frac{U^2}{4} \frac{1}{\omega} \delta_{ab} \delta_{\sigma\sigma'} + \frac{U^2}{4} \frac{\chi^{ab\sigma\sigma'}(k)}{\omega^2} \tag{16}$$

for the half-filled case. $\chi^{ab\sigma\sigma'}(k)$ is given by

$$\begin{aligned}\chi^{ab\sigma\sigma'}(k) = & -\varepsilon_k^{ab\sigma\sigma'} + \frac{2}{N}\sum_q \varepsilon_q^{ab\sigma\sigma'}\delta_{ab}\delta_{\sigma\sigma'} + \frac{2}{N}\sum_q \varepsilon_q^{ab\sigma\sigma'}\delta_{ab}\delta_{\sigma,-\sigma'} \\ & - \frac{4}{N^2}\sum_{qlj} \varepsilon_q^{ab\sigma\sigma'} e^{i(k-q)(r_l-r_j)} \left\langle c^\dagger_{la-\sigma} c^\dagger_{jb-\sigma'} c_{la-\sigma} c_{jb-\sigma'} \right\rangle \\ & - \frac{4}{N^2}\sum_{qlj} \varepsilon_q^{ab\sigma\sigma'} e^{i(k-q)(r_l-r_j)} \left\langle c^\dagger_{la-\sigma} c^\dagger_{jb\sigma'} c_{la\sigma} c_{jb-\sigma'} \right\rangle\end{aligned} \tag{17}$$

where N is a normalization factor. Corrections to this expression depend either on the double occupations or on the expectation value of hopping terms. As shown in section Supplementary Note 1 C these are much smaller then the expectation values in $\chi^{ab\sigma\sigma'}(k)$.
For on-site terms (with $a = b$) the expectation values in the last two terms vanish and we arrive at

$$\chi^{ab\sigma\sigma'}_{\text{on-site}} = -\varepsilon^{ab\sigma\sigma'}_{\text{on-site}}\delta_{ab} + \frac{2}{N}\sum_q \varepsilon_q^{ab\sigma\sigma'}\delta_{ab}\delta_{\sigma\sigma'} + \frac{2}{N}\sum_q \varepsilon_q^{ab\sigma\sigma'}\delta_{ab}\delta_{\sigma,-\sigma'} \tag{18}$$

which under the assumption that all diagonal q-dependent terms in $\varepsilon_q^{ab\sigma\sigma'}$ sum to zero reduces to

$$\chi^{ab\sigma\sigma'}_{\text{on-site}} = \varepsilon^{ab\sigma\sigma'}_{\text{on-site}}\delta_{ab}. \tag{19}$$

This is consistent with what one would expect for on-site terms. Those can be exactly described within the atomic limit, leading only to constant shifts of poles and zeros respectively. Going beyond the atomic limit we therefore would not expect any renormalization.
For the remaining terms we have to evaluate the expectation values instead:

$$\begin{aligned}\chi^{ab\sigma\sigma'}_{\text{rest}}(k) = & -\varepsilon_k^{ab\sigma\sigma'} - \frac{4}{N^2}\sum_{qlj} \varepsilon_q^{ab\sigma\sigma'} e^{i(k-q)(r_l-r_j)} \left\langle c^\dagger_{la-\sigma} c^\dagger_{jb-\sigma'} c_{la-\sigma} c_{jb-\sigma'} \right\rangle \\ & - \frac{4}{N^2}\sum_{qlj} \varepsilon_q^{ab\sigma\sigma'} e^{i(k-q)(r_l-r_j)} \left\langle c^\dagger_{la-\sigma} c^\dagger_{jb\sigma'} c_{la\sigma} c_{jb-\sigma'} \right\rangle.\end{aligned} \tag{20}$$

The prefactor in front of the expectation values in Eq. (20) restricts j and l to sites connected by a hopping in the non-interacting dispersion. This can be seen by defining $\alpha = l - j$ and explicitly writing the dispersion as a sum over all hopping directions β:

$$\begin{aligned}\frac{4}{N^2}\sum_{q,l,j} \varepsilon_q^{ab\sigma\sigma'} e^{i(k-q)(r_l-r_j)} f_{lj}^{ab\sigma\sigma'} & = \frac{4}{N}\sum_{q,\alpha,\beta} t_\beta^{ab\sigma\sigma'} e^{iqd_\beta} e^{i(k-q)d_\alpha} f_\alpha^{ab\sigma\sigma'} \\ & = 4\sum_\beta t_\beta^{ab\sigma\sigma'} e^{ikd_\beta} f_\beta^{ab\sigma\sigma'}\end{aligned} \tag{21}$$

where f stands for either one of the expectation values in Eq. (20) and d_α indicates the distance between two sites with indices differing by α. Here it is assumed that $d_\beta \neq 0$ or $a \neq b$, otherwise the corresponding expectation values vanish (as already discussed for the on-site terms).
Considering the restrictions for l and j, the first expectation value can be rewritten as $\left\langle c^\dagger_{la-\sigma} c^\dagger_{jb-\sigma'} c_{la-\sigma} c_{jb-\sigma'} \right\rangle = -\langle n_{la-\sigma} n_{jb-\sigma'}\rangle$. Now we define the correlation function $\Delta^{ab\sigma\sigma'}_{lj\sigma} \equiv \Delta^{ab\sigma\sigma'}_{l-j}$ which fulfills

$$\langle n_{l,a,-\sigma} n_{j,b,-\sigma'}\rangle = \langle n_{l,a,-\sigma}\rangle \langle n_{j,b,-\sigma'}\rangle - \Delta^{ab\sigma\sigma'}_{lj\sigma} = \frac{1}{4} - \Delta^{ab\sigma\sigma'}_{lj\sigma}. \tag{22}$$

As shown in section Supplementary Note 1 C the second expectation value can be expressed using the same correlation function:

$$\left\langle c^\dagger_{la-\sigma} c^\dagger_{jb\sigma'} c_{la\sigma} c_{jb-\sigma'} \right\rangle = 2\Delta^{ab\sigma\sigma'}_{lj\sigma}. \tag{23}$$

Inserting the results into Eq. (20) we get

$$\chi_{\text{rest}}^{ab\sigma\sigma'}(k) = -12 \sum_{\beta} t_{\beta}^{ab\sigma\sigma'} e^{ikd_\beta} \Delta_{\beta}^{ab\sigma\sigma'} \tag{24}$$

which is just the renormalized non-interacting dispersion. As will be shown in the section below for certain limits of the parameters the correlation function can be determined analytically, yielding

$$\Delta_{\beta}^{ab\sigma\sigma'} = \frac{1}{4} \frac{(t^{ab\sigma\sigma'})^2 \beta}{U} \tag{25}$$

which leads to a renormalization factor of $3\frac{(t^{ab\sigma\sigma'})^2\beta}{U}$, consistent with the the results of Pairault et al. [10] for a single-orbital cosine-dispersion.
The full self-energy is given by

$$\Sigma_{ab\sigma\sigma'}(\omega, k) = \frac{U}{2}\delta_{ab} + \left(\frac{U^2/4}{\omega - \chi_{\text{rest}}(k) - \chi_{\text{on-site}}} \right)_{ab\sigma\sigma'} = \frac{U}{2}\delta_{ab} + \left(\frac{U^2/4}{\omega + \widetilde{H}_0(k)} \right)_{ab\sigma\sigma'} \tag{26}$$

where $\widetilde{H}_0(k)$ has been identified with $-\chi_{\text{rest}}(k) - \chi_{\text{on-site}}$. Note that $\chi_{\text{rest}}(k)$ has the opposite sign in respect to the non-interacting Hamiltonian terms while $\chi_{\text{on-site}}$ has the same sign.

C. Correlation values

As discussed above the result of the high-frequency expansion depends crucially on the correlation value $\langle n_{i\sigma} n_{j\sigma} \rangle$. In order to justify the above result for the analytic expression for the self-energy, in this section we provide numerical and analytic evidence that $\langle n_{i\sigma} n_{j\sigma} \rangle$ does indeed behave as assumed in the previous section.

1. *Expansion about the atomic limit*

In order to estimate Δ_{lj} we use an expansion of the thermal expectation value $\left\langle \hat{O} \right\rangle = \frac{1}{Z} \sum_n \langle n| \exp(-\beta H + \beta \mu N) \hat{O} |n\rangle$. The chemical potential term commutes with H and we can ignore it. After rewriting the exponential function as a series,

$$\exp(-\beta H) = \sum_x \frac{(-\beta H)^x}{x!} = \sum_x \frac{(-\beta)^x}{x!} (H_U + H_t)^x, \tag{27}$$

we restrict ourselves to the lowest order terms in t:

$$(H_U + H_t)^x = H_U^x + \sum_{a+b=x-1} H_U^a H_t H_U^b + \sum_{a+b+c=x-2} H_U^a H_t H_U^b H_t H_U^c + \mathcal{O}(H_t^3). \tag{28}$$

The U and t subscripts correspond to the interaction and hopping parts of the Hamiltonian respectively. For $\hat{O} = n_{l\sigma} n_{j\sigma}$ the first order in t vanishes and we get

$$\exp(-\beta H) = \sum_x \frac{(-\beta U)^x}{x!} \left(h_U^x + \Theta(x-2) \frac{t^2}{U^2} \sum_{a+b+c=x-2} h_U^a h_t h_U^b h_t h_U^c + \mathcal{O}(t^4) \right) \tag{29}$$

where $H_U = U h_U$ and $H_t = t h_t$ and the second term contributes only for $x \geq 2$. We can now make use of eq. (29) in the expression for the expectation value, with particular attention posed to the effect of the series of operators defined by h_U, h_t and $\hat{O}$ on the number of doubly-occupied sites in the system. In this sense, it can be shown that at large βU the contribution of each state to the sum is suppressed in a way proportional to the number of double occupations. In first approximation, therefore, we are allowed to restrict our further analysis to states with no double occupations. The final step is to determine the contributing states in the trace, by counting how many hopping processes defined by Eq. (29) are allowed for a given electronic configuration. The result depends on the relation between i and j:

$$\langle n_{l\sigma} n_{j\sigma} \rangle = \frac{1}{4} \frac{1 + \frac{t^2}{U^2} \beta U (\frac{z}{2} N - \delta_{<lj>})}{1 + \frac{t^2}{U^2} \beta U \frac{z}{2} N} \tag{30}$$

where $\delta_{<lj>}$ is non-zero only if l and j are neighbors (or, more generally, if there is a hopping term connecting l and j). The factor $\frac{z}{2}N$ and the correction $\delta_{<lj>}$ comes from the number of states with non-zero contribution in the sum over all possible states. Here z is the coordination number and N is the number of sites. We evaluate this expression for small t keeping in mind that the number of sites N is large. Eq. (30) can then be seen as the expansion of

$$\frac{1}{4}\left(\frac{1+\frac{t^2}{U^2}\beta U(\frac{z}{2}-\frac{\delta_{<lj>}}{N})}{1+\frac{t^2}{U^2}\beta U\frac{z}{2}}\right)^N \approx \frac{1}{4}\left(1-\frac{t^2}{U^2}\beta U\frac{\delta_{<lj>}}{N}\right)^N \approx \frac{1}{4}-\frac{1}{4}\frac{t^2}{U}\beta\delta_{<lj>}. \tag{31}$$

Thus we can infer that $\Delta_{lj} = \frac{1}{4}\frac{t^2}{U}\beta\delta_{<lj>}$. This result is restricted to $\frac{t^2\beta}{U} \ll 1$ and $\beta U \gg 1$ due to the assumptions needed in the expansion. For the expectation value of the spin-hopping term $\left\langle c^\dagger_{la-\sigma}c^\dagger_{jb\sigma'}c_{la\sigma}c_{jb-\sigma'}\right\rangle$ a similar analysis can be done. For the second-order term the two hopping terms coming from the expansion in Eq. (29) have to compensate the spin-hopping. This is only possible if l and j are connected by a hopping in the non-interacting dispersion, yielding $\left\langle c^\dagger_{la-\sigma}c^\dagger_{jb\sigma'}c_{la\sigma}c_{jb-\sigma'}\right\rangle = 2\frac{1}{4}\frac{t^2}{U}\beta\delta_{<lj>}$ where the factor of 2 is due to the possibility to interchange the two hopping terms.

2. *Numerical results*

In order to check the analytic results we used exact diagonalization (ED) calculations for a one dimensional chain with periodic boundary conditions. As shown in Supplementary Figure 1 the dependence of the correlation value on t, β and U predicted by the analytic calculations of the previous section is very well reproduced (for small t, small β and large U respectively). Further the double occupation and the expectation value of a hopping term are much smaller, justifying to neglect corrections including these expressions in the derivation for the self-energy.

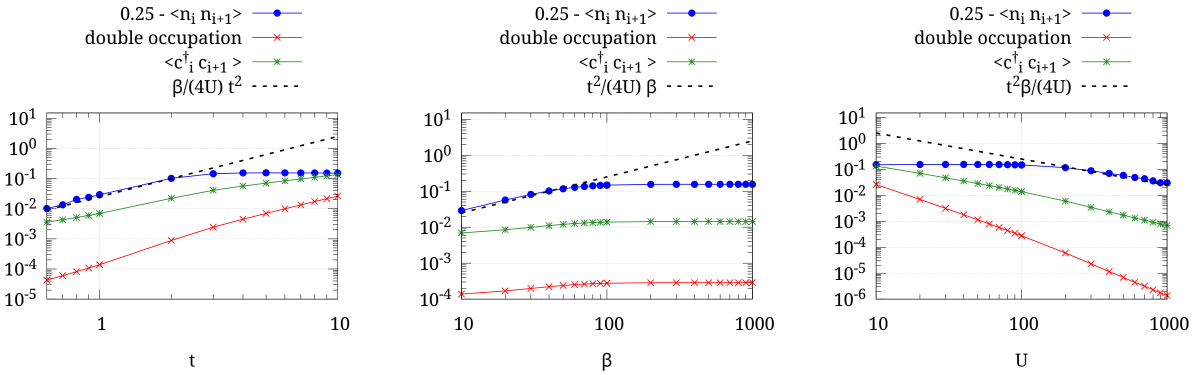

Supplementary Figure 1: ED results for different expectection values as a function of hopping t, inverse temperature β and interaction U. For the leftmost plot $\beta = 10$ and $U = 100$; for the center plot $t = 1$ and $U = 100$; and for the right hand plot $t = 1$ and $\beta = 100$.

D. Analytic estimation of higher-order contributions

In order to demonstrate that higher-order corrections to Eq.26 are suppressed for large U, we model two Hubbard bands using a continued fraction representation. We construct a spectral function with a gap of size $U - W$ (where $W = 4t$ is the bandwidth of each Hubbard band) and each of the two Hubbard bands is parametrized with a continued fraction, as for one hole in the t-J model (see Refs.[11, 12]):

$$G^{J}_{\text{band}}(\omega) = \cfrac{1}{\omega - \cfrac{t^2}{\omega - \frac{J}{2} - \cfrac{t^2}{\omega - 2\frac{J}{2} - \cfrac{t^2}{\omega - 3\frac{J}{2} - \dots}}}}. \tag{32}$$

We also introduce an overall energy shift ϵ, in order to have the flexibility of moving the position of the self-energy pole. The overall Green's function can then be written as

$$G(\omega) = \frac{1}{2}\left(G^{J_1}_{\text{band}}\left(\omega - \frac{U}{2} - \epsilon + i\delta\right) - G^{J_2}_{\text{band}}\left(-\omega - \frac{U}{2} + \epsilon - i\delta\right)\right). \tag{33}$$

where a mismatch between the two Hubbard bands is introduced via J_1 and J_2 so that we can additionally break particle-hole symmetry. With particle-hole symmetry, the low-energy poles would not be effected by U at any order. In Supplementary Fig. 2 we show an example of $G(\omega)$ with different shapes of the Hubbard bands.

The crucial point is that it is possible to write this sum again as a continued fraction. The coefficients can be compared, order by order in an expansion for large frequencies:

$$G(\omega) = \cfrac{1}{\omega - \epsilon - \cfrac{\eta_1}{\omega - \tilde{\epsilon}_1 - \cfrac{\rho_1}{\omega - \tilde{\epsilon}_2 - \cfrac{\eta_2}{\omega - \tilde{\epsilon}_3 - \cfrac{\rho_2}{\omega - \dots}}}}} \tag{34}$$

with

$$\begin{aligned}
\eta_1 &= \frac{U^2}{4} + t^2 \\
\eta_2 &= \frac{U^2}{4} + \frac{U}{4}(J_1 + J_2) + \frac{J_1 J_2}{4} + \frac{-J_1^3 + J_1^2 J_2 + J_1 J_2^2 - J_2^3 - 24 J_1 t^2 - 24 J_2 t^2}{32U} + \alpha_1 \mathcal{O}\left(\frac{t^2}{U^2}\right) \\
\rho_1 &= 4t^2 + \frac{2t^2(J_1 + J_2)}{U} + \alpha_2 \mathcal{O}\left(\frac{t^2}{U^2}\right) \\
\rho_2 &= 4t^2 + \frac{7t^2(J_1 + J_2)}{U} + \alpha_3 \mathcal{O}\left(\frac{t^2}{U^2}\right)
\end{aligned} \tag{35}$$

and

$$\begin{aligned}
\tilde{\epsilon}_1 &= \epsilon + \frac{(J_1 - J_2)t^2}{4t^2 + U^2} \\
\tilde{\epsilon}_2 &= \epsilon + \frac{(J_1 - J_2)}{2} + \frac{J_1^2 - J_2^2}{16U} + \alpha_4 \mathcal{O}\left(\frac{t^2}{U^2}\right) \\
\tilde{\epsilon}_3 &= \epsilon - \frac{(J_1^2 - J_2^2)}{16U} + \alpha_5 \mathcal{O}\left(\frac{t^2}{U^2}\right)
\end{aligned} \tag{36}$$

Here α_i are prefactors which ensure the correct physical dimension of the higher-order corrections. Substituting $t = 0$ and $J_1 = J_2 = 0$ into these coefficients keeping U finite, we see that Eq. 34 correctly reduces to Green's function of the atomic limit with only two floors of the continuous fraction ($\rho_1 = 0$).

In the general case, the self-energy can be immediately determined from Eq. 34:

$$\Sigma(\omega) = \cfrac{U^2/4 + t^2}{\omega - \tilde{\epsilon}_1 - \cfrac{\rho_1}{\omega - \tilde{\epsilon}_2 - \cfrac{\eta_2}{\omega - \tilde{\epsilon}_3 - \cfrac{\rho_2}{\omega - \ldots}}}} \tag{37}$$

For large values of U the low-energy poles of the self-energy are given by the solution of

$$\omega - \tilde{\epsilon}_1 + \frac{16t^2(\omega - \epsilon)}{U^2} + \frac{(J_1 + J_2)t^2(J_1 - J_2 - 8(\omega - \epsilon))}{U^3} + \alpha_6 \mathcal{O}\left(\frac{1}{U^4}\right) = 0, \tag{38}$$

which leads to

$$\omega = \tilde{\epsilon}_1 + \alpha_7 \mathcal{O}\left(\frac{t^2}{U^2}\right), \tag{39}$$

demonstrating the suppression of higher orders for poles of the self-energy inside the gap and confirming the applicability of Eq.(26). In fact, as shown in Supplementary Fig.2 the higher orders have an influence only on the outer structure of the Hubbard bands. Here, $\tilde{\epsilon}_1$ which determines the position of the poles also includes corrections which are suppressed by large U, in addition to the correction which are a consequence of higher orders on the continued fraction. Note that the example presented here corresponds to the case where ϵ is an on-site energy, thus the poles are located at $+\epsilon$.
For a numerical demonstration of the suppression of higher orders for specific models see Sec.Supplementary Note 3 E.

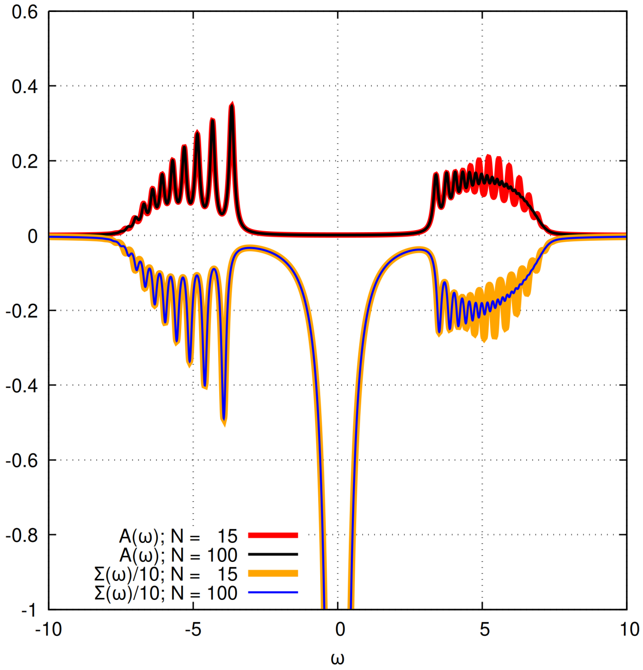

Supplementary Figure 2: Spectral function and corresponding self-energy for a Mott insulator made of two Hubbard bands individually described by continued fractions (see Eq.(33) with $\epsilon = 0$, $t = 1$, $J_1 = 0.2$, $J_2 = 0.5$ and $U = 10$) truncated at order N.

E. Topological invariants from the zeros

Here, we present general results for the topological invariant [13][14, 15]

$$Q[G] = \frac{1}{6}\int \frac{d^2p d\omega}{2\pi \mathrm{Vol(BZ)}} e^{i\omega 0^+} q[G],$$
$$q[G] = \epsilon_{\mu\nu\rho}\, \mathrm{tr}\big[(G^{-1}\partial_\mu G)(G^{-1}\partial_\nu G)(G^{-1}\partial_\rho G)\big]. \tag{40}$$

We use a Wiegmann-Polyakov like relationship, which can be straightforwardly checked

$$q[G_1G_2] = q[G_1] + q[G_2] + 2\epsilon_{\mu\nu\rho}\partial_\mu\, \mathrm{tr}\big[(G_1\partial_\nu G_1^{-1})(G_2\partial_\rho G_2^{-1})\big]. \tag{41}$$

Thus, assuming sufficiently quickly decaying Green's functions, the boundary conribution drops out and $Q[G_1G_2] = Q[G_1] + Q[G_2]$. Next, we use that for our Ansatz, Eq. (26), we can write $G^{-1} = \tilde{G}\mathcal{G}_H^{-1}$ with

$$\tilde{G}^{-1} = i\omega + \tilde{H}$$
$$\mathcal{G}_H^{-1} = (i\omega + \tilde{H})(i\omega - H) - U^2/4, \tag{42}$$

where $\mathcal{G}_H$ accounts for Hubbard bands and $\tilde{G}$ for the zeros. We thus find for the invariant

$$Q[G] = -Q[\tilde{G}] + Q[\mathcal{G}_H] \overset{\text{see below}}{=} -Q[\tilde{G}]. \tag{43}$$

We explain below that $Q[\mathcal{G}_H] = 0$, such that in the Mott insulating regime, the topological invariant is given by the band topology of $\tilde{H}$ instead of H (note that the reverse sign in front of $\tilde{H}$ in the definition of $\tilde{G}$ as opposed to the sign of H in the usual definition of the Green's function, as well as the reverse sign in Eq. (43): If $H = \tilde{H}$, $Q[G]$ is the same for $U = 0$ and large U topological Mott insulation.)

What is left is to show the vanishing contribution of Hubbard bands. It is convenient to use the notations $H = \bar{H} + \Delta H/2$ and $\tilde{H} = \bar{H} - \Delta H/2$, so that

$$\mathcal{G}_H^{-1} = (i\omega - \Delta H/2)^2 + [\Delta H, \bar{H}]/2 - [\bar{H}^2 + U^2/4]. \tag{44}$$

First, consider the case $\Delta H = 0$ (i.e. $H = \tilde{H}$) in which the poles of $\mathcal{G}_H$ are given by $i\omega = \pm\sqrt{\epsilon_n^2 + U^2/4}$, where $\bar{H}\,|n\rangle = \epsilon_n\,|n\rangle$ is the single-particle eigenbasis of $\bar{H}$. Despite the fact that the regulator $e^{i\omega 0^+}$ constrains the contribution to negative Hubbard bands only, both negative $\epsilon_n < 0$ and positive $\epsilon_n > 0$ bands of the non-interacting Hamiltonian contribute. While each band might contribute a non-trivial topological invariant, the overall sum over all bands $|n\rangle$ must vanish, hence $Q[\mathcal{G}_H] = 0$.

Next, we consider non-zero ΔH. In the limit of small corrections (on the scale of U), and given that the topological invariant can not smoothly change, we can conclude that there is a finite environment around $\Delta H = 0$ in which $Q[\mathcal{G}_H] = 0$ still holds. One way to see this is to simply Taylor expand in ΔH, which is legitimate since there is a gap of order U.

F. Numerical topological invariant

For an interacting system the topological invariant can be computed numerically as described in Ref.[14, 16, 17]. The result for a Chern insulator (see Sec.Supplementary Note 3 J) is shown in Supplementary Fig. 3 for different values of a generic renormalization α of the non-local part of the Hamiltonian in the denominator of the self-energy (Eq.(26)). The region of non-trivial behavior decreases when α is reduced, up to the point where the system is always trivial for $\alpha = 0$ which corresponds to a single-site DMFT self-energy. The positions of the topological transitions are consistent with an analysis of the renormalized Hamiltonian as shown in the previous section.

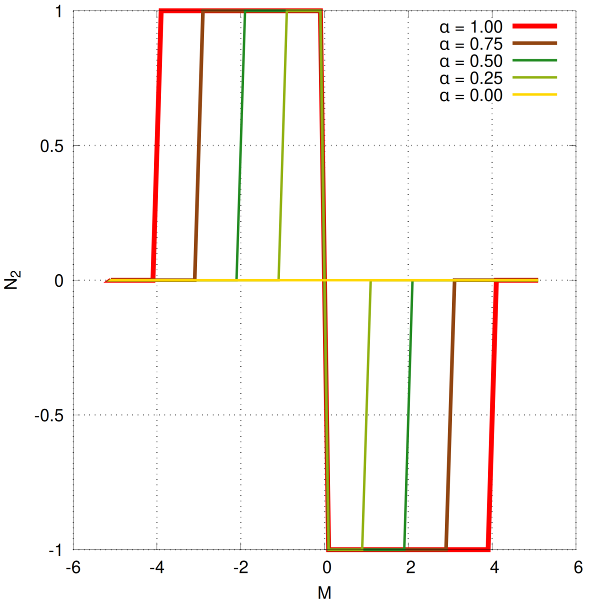

Supplementary Figure 3: Topological invariant for a Chern insulator (see Eq.(58) with $t = 1.0$ and $\lambda = 0.3$) in the Mott phases ($U = 16$) modeled using the analytic formula for the self-energy in Eq.26. The renormalization of the non-local terms is given by α.

G. Application of the analytic self-energy formula to the Hubbard dimer

Applying the analytic self-energy formula to a finite system means replacing the k-dependence with the full matrix structure of the Hamiltonian H_0 in real space:

$$\Sigma(\omega) = \frac{U^2/4}{\omega + \alpha H_0}. \tag{45}$$

Here, the renormalization is taken into account by the prefactor α.
For the Hubbard dimer the full Hamiltonian H is given by

$$H = -t \sum_{\sigma} \left(c^{\dagger}_{1\sigma} c_{2\sigma} + c^{\dagger}_{2\sigma} c_{1\sigma} \right) + U \sum_{i=1,2} n_{i\uparrow} n_{i\downarrow} - \frac{U}{2} \sum_{i=1,2;\sigma} n_{i\sigma}. \tag{46}$$

In the following the high-frequency approach is tested against the exact solution for the Hubbard dimer, both by applying Eq.(13) and by explicitly solving the high-frequency expansion for the dimer, i.e. solving Eq.(9) with the full dimer Hamiltonian.
Furthermore it is possible to describe the interface of two SSH models using a dimer where U is restricted to one site.

1. Exact solution

At zero temperature the exact self-energy is given by [18]

$$\begin{aligned} \Sigma_{11} &= \frac{U^2}{4} \left(\frac{\omega}{\omega^2 - 9t^2} \right) \\ \Sigma_{12} &= \frac{U^2}{4} \left(\frac{3t}{\omega^2 - 9t^2} \right), \end{aligned} \tag{47}$$

Expanding in orders of $\frac{1}{\omega}$ leads to

$$\begin{aligned} \Sigma_{11} &= \frac{U^2}{4\omega} + \frac{9U^2t^2}{4\omega^3} + \mathcal{O}\left(\frac{1}{\omega^5} \right) \\ \Sigma_{12} &= \frac{3U^2t}{4\omega^2} + \frac{27U^2t^3}{4\omega^4} + \mathcal{O}\left(\frac{1}{\omega^6} \right). \end{aligned} \tag{48}$$

2. Analytic formula

With

$$H_0 = \begin{pmatrix} 0 & -t \\ -t & 0 \end{pmatrix} \tag{49}$$

and $\alpha = 3$ (due to $\langle n_{i\sigma} n_{j\sigma} \rangle = 0$ leading to $\Delta_{ij} = \frac{1}{4}$) Eq.(45) yields the same self-energy as the exact solution.

3. Explicit high-frequency expansion

For the high-frequency expansion expectation values have to be evaluated (Eq. 9). This can also be done by explicitly carrying out the calculations for the dimer Hamiltonian. Then it is enough to know that the ground state has the form

$$|\psi_0\rangle = \gamma \left|\uparrow\downarrow\right\rangle - \gamma \left|\downarrow\uparrow\right\rangle + \beta \left|0 \uparrow\downarrow\right\rangle + \beta \left|\uparrow\downarrow 0\right\rangle \tag{50}$$

with $\gamma^2 + \beta^2 = \frac{1}{2}$. Using this assumptions, we can confirm that the first four orders in $\frac{1}{\omega}$ are equivalent to Eq. (48).

4. Hubbard dimer as interface

By restricting the Hubbard interaction to one site and changing the coupling between the sites we can model the interface between two SSH chains (one interacting, one non-interacting) in the atomic limit. For the analytic formula the interaction is restricted to one site by setting all other elements of the self-energy to zero after the inversion:

$$\Sigma(\omega) = \begin{pmatrix} \frac{U^2}{4}\frac{1}{\omega - \frac{\alpha^2 t^2}{\omega}} & 0 \\ 0 & 0 \end{pmatrix}. \tag{51}$$

As shown in Supplementary Figure 4 the agreement with ED results is perfect. This simple example captures the zero/pole annihilation upon coupling the two atoms. Furthermore the difference between the "absence of weight" and a zero becomes clear: the weight of annihilated zeros and poles is orders of magnitude larger than the zeros but still orders of magnitude smaller than the poles.

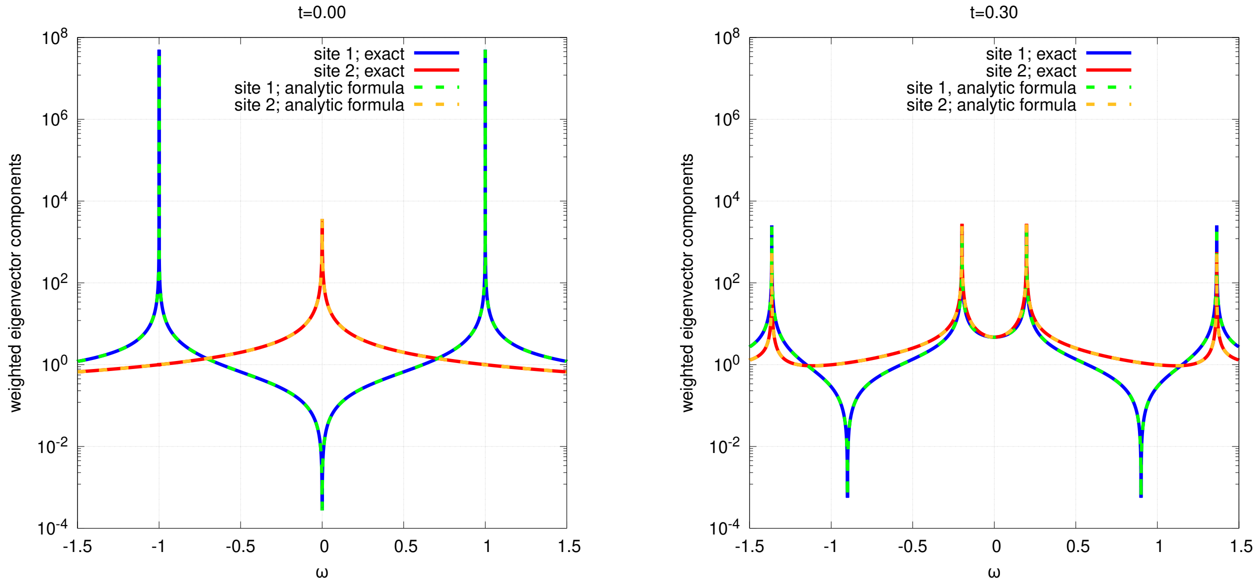

Supplementary Figure 4: Comparison of the exact solution and the prediction of the analytic formula for the self-energy (Eq.(51)) for an uncoupled Hubbard dimer (left) and a coupled Hubbard dimer (right) with $U = 2$ only on site 1. See Methods in main text for a description of the weighted eigenvector components.

Supplementary Note 2. SLAB CALCULATIONS USING THE ANALYTIC FORMULA

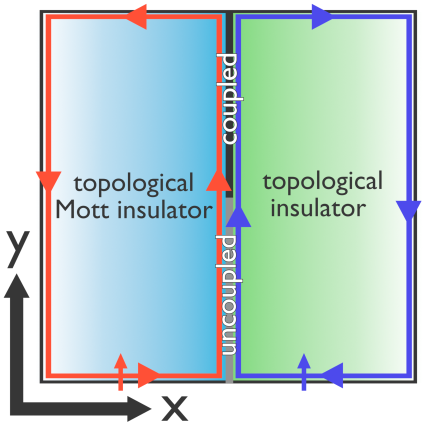

Supplementary Figure 5: Slab geometry consisting of an interface between two topological insulators, one with $U = 0$, the other in the Mott phase. Along the gray edge of the interface there is no coupling between the two sides, while a finite coupling exists along the black edge. The blue and red lines indicate the edge states and zeros respectively and are only shown for one spin species, the other having all directions inverted. At the interface the edge channels have the same direction which is a consequence of the plus sign in the denominator of Eq. (1) in the main text.

For the slab calculations we use a geometry as depicted in Supplementary Fig. 5. The right hand side is a non-interacting topological insulator, whereas the the left hand side is either a trivial or topological Mott insulator. We compute the Green's function using

$$G = (\omega\mathbb{1} - H_0 - \Sigma(\omega))^{-1} \tag{52}$$

where the non-interacting Hamiltonian is given by a BHZ model[19–21]:

$$H_0 = \sum_{i\alpha\sigma} M\tau^z_{\alpha\alpha} c^\dagger_{i\alpha\sigma} c_{i\alpha\sigma} - \sum_{i\alpha\beta\mu\sigma} t^{\alpha\beta}_{\mu\sigma} c^\dagger_{i+\mu\alpha\sigma} c_{i\beta\sigma} \tag{53}$$

with

$$t_{\mu\sigma} = \begin{pmatrix} t & \sigma\frac{i}{2}\lambda e^{i\sigma\theta_\mu} \\ \sigma\frac{i}{2}\lambda e^{-i\sigma\theta_\mu} & -t \end{pmatrix} \tag{54}$$

where μ runs over the the neighbors ($\pm x$,$\pm y$), θ_μ is the angle between the x-axis and direction of the neighbor and σ is the spin. The self-energy is computed using the analytic formula (Eq. (26)). In this case we do not have a k-dependence. Instead the full matrix H_0 (with orbital, spin and spatial degrees of freedom) is taken into account. The renormalization of the non-local terms is set to one for simplicity. Due to the stability of topological edge states, the exact parameters of the system aren't important as long as there is no topological phase transition. Therefore we can essentially choose the parameter almost arbitrarily as long as we stay in the topological phase because our aim is to see the effect of the interplay of edge poles and zeros. The M-parameters are chosen with opposite signs for the two slab sides in order to ensure that the zeros and poles can annihilate. The restriction of U to one side of the slab is implemented by setting the components of the self-energy corresponding to a non-interacting site to zero after the inversion.

At the interface the coupling is set to zero on the gray part and to one on the black part. The wave packet is initialized (restricted to one spin species) using a gaussian on the uncoupled egde (gray):

$$\psi(r,t) = \frac{1}{N}\int dr' G(r,r',t) e^{-\left((r_{0,x}-r'_x)^2/s_x^2 + (r_{0,y}-r'_y)^2/s_y^2\right)/2}. \tag{55}$$

Here r and r' are the slab coordinates and r_0 is the point where the packet is initialized with variance s_x and s_y in x- and y-direction respectively. N is a normalization factor.

Supplementary Note 3. ADDITIONAL RESULTS

A. QMC vs ED for the SSH model

As a check of the ED calculations we compare the results for a finite SSH chain with quantum Monte Carlo (QMC) (Supplementary Figure 6). In order to avoid an analytic continuation of the QMC data, we use the ED results on the imaginary axis. As the figure is showing we find a very good agreement between the two methods and also with the results obtained using the analytic formula. Especially these results are showing that the analytic approach is viable (at least qualitatively) for a wide range of temperatures, starting from the zero temperature limit where ED is capable of describing the system, to larger temperatures, accessible using QMC. With QMC we are also able to investigate much larger chains. The results are shown in Supplementary Fig.7, confirming that our results are stable also for large systems.

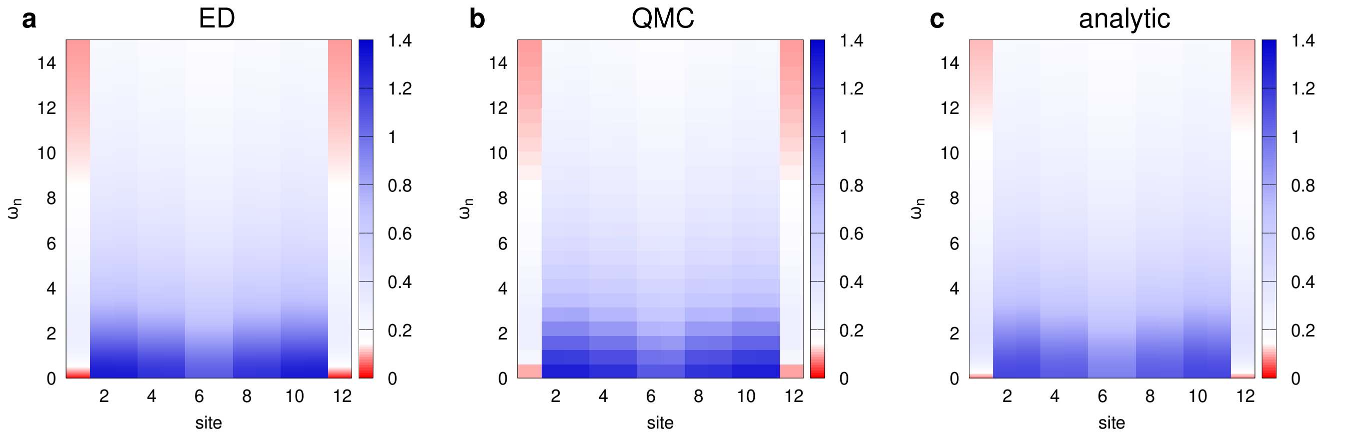

Supplementary Figure 6: Comparison between ED (a), QMC (b) and the analytic self-energy formula (c) for a non-trivial finite SSH chain on the Matsubara axis. The interaction is $U = 4$ and the hopping parameters are $v = 0.1$ and $w = 1.0$. The QMC results are for $\beta = 10$, the ED calculations are at zero temperature (where the spacing of the Matsubara frequencies is set to be equivalent to $\beta = 1000$). For the analytic formula the spacing of the Matsubara frequencies is set to $\beta = 100$ and the renormaliazion of the parameters to 3.

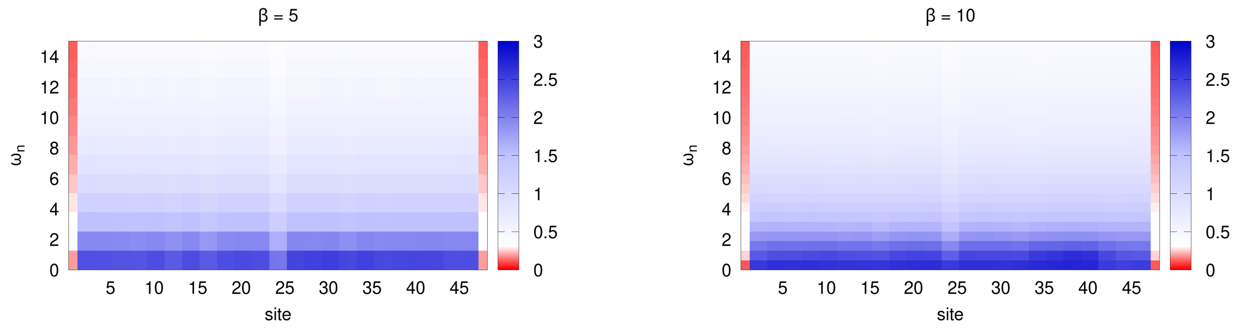

Supplementary Figure 7: QMC results for a SSH chain with 48 sites for $U = 4$. The inverse temperature is given by $\beta = 5$ and $\beta = 10$ respectively. The SSH model is in the topological phase with parameters $v = 0.1$ and $w = 1.0$.

B. Comparison of analytic formula and numerical results for an interface between SSH chains

Supplementary Figure 8 shows that we find a very good agreement between ED and the analytic formula for the self-energy (Eq. 1 in the main text) for the coupled and uncoupled interface of two SSH chains (one in the Mott phase, the other non-interacting), that have been discussed in the main text in Supplementary Figure 3. The renormalization parameter for the analytic formula has been chosen as $\alpha = 3$.

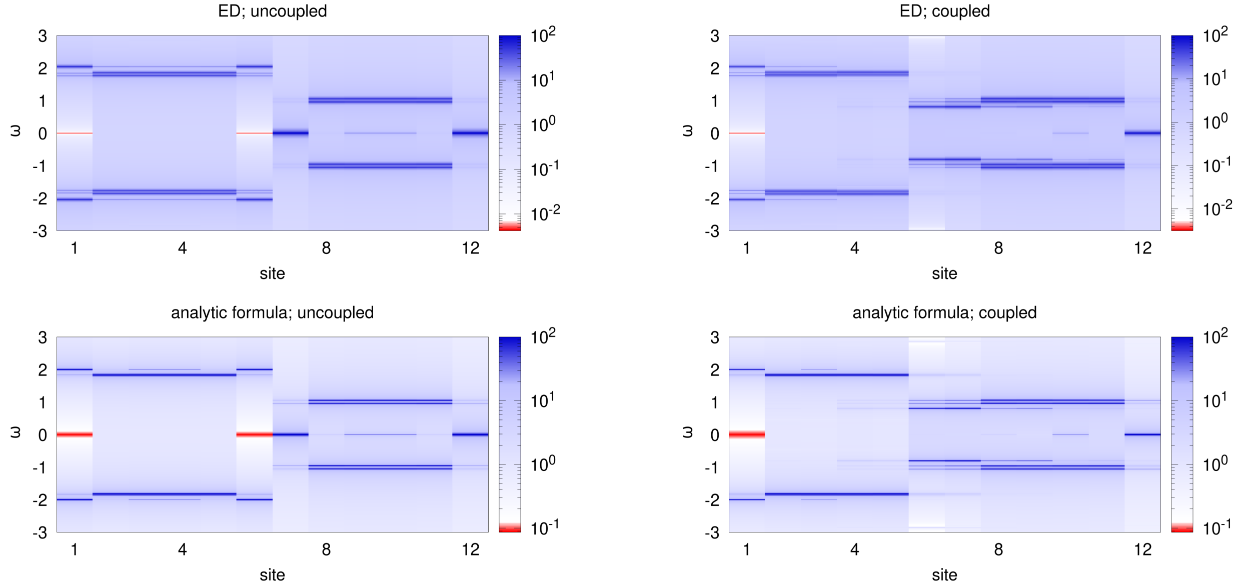

Supplementary Figure 8: Comparison between ED (top) and the analytic formula (Eq.(26)) (down) for uncoupled SSH chains (left) and coupled SSH chains (right). The right chain in each plot is for $U = 0$ and the left chain for $U = 4$.

C. SSH model with broken chiral symmetry

Adding on-site terms to the SSH model breaks the chiral symmetry. As a consequence the edge states (and zeros) are not fixed to zero energy. Nonetheless we still find an annihilation at an interface as shown in Supplementary Fig. 9 and Supplementary Fig. 10 even when the energies of pole and zero are not the same. This is required by the topology because both sides of the chain are in the same topological phase, hence at an interface no protected, gapless state can appear.
Let us note in passing that without introducing such local mismatch but considering instead U only on one half of the chain and compensating the Hartree contribution with the chemical potential, as we do in the main text, does not break the chiral symmetry as it preserves time-reversal as well as the particle-hole symmetry.

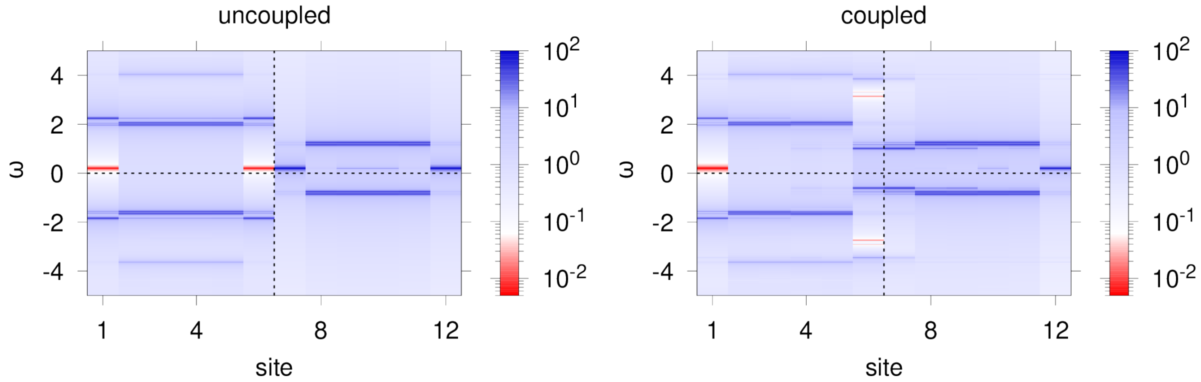

Supplementary Figure 9: ED results for finite chain SSH model interfaces (uncoupled on the left and coupled on the right) with $U = 4$ only on the left part of the chain and an overall on-site energy shift of 0.2. The color scale corresponds to weighted eigenvector components (see Methods in main text).

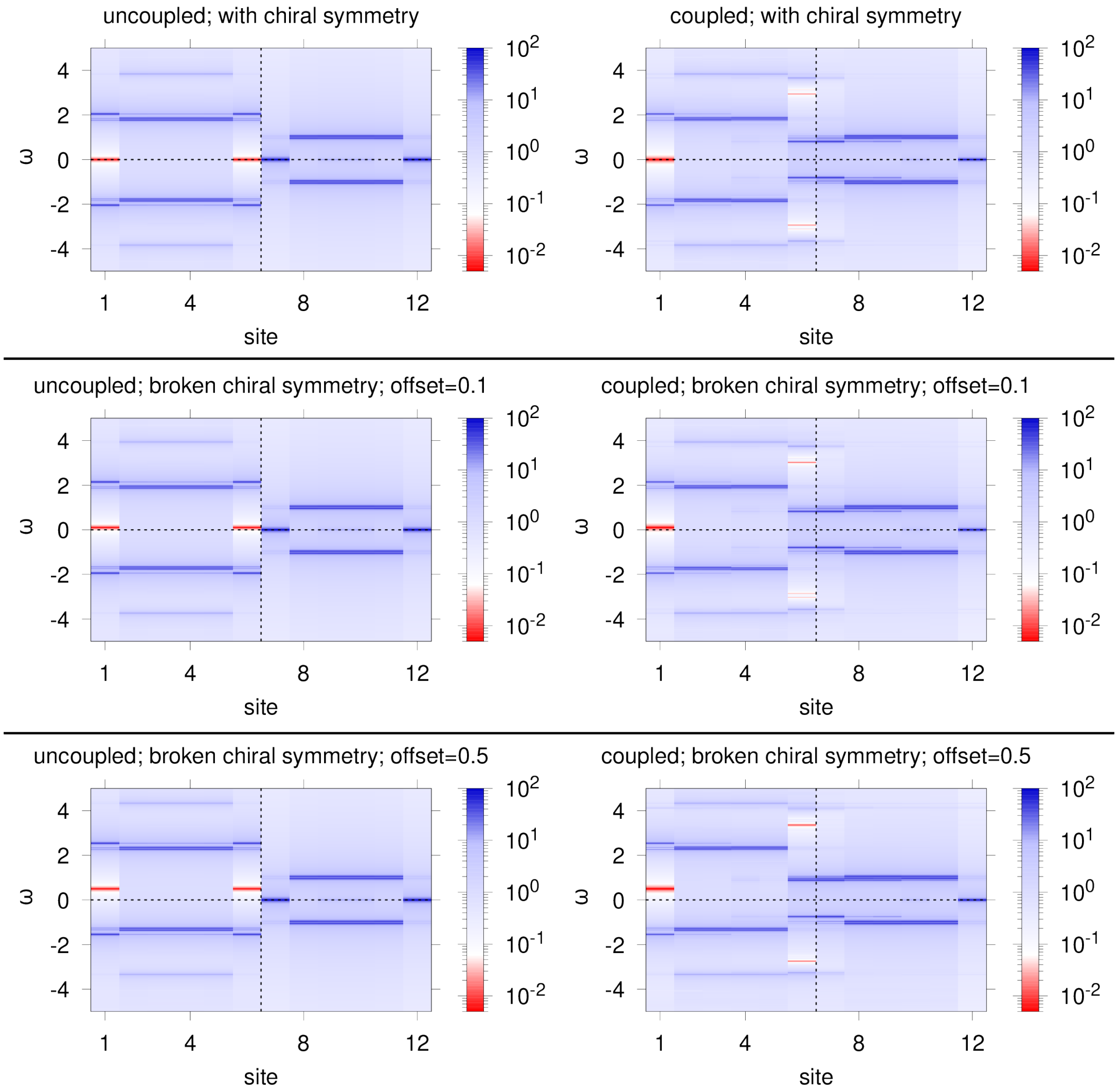

Supplementary Figure 10: ED results for finite chain SSH model interfaces with $U = 4$ only on the left part of the chain. The color scale corresponds to weighted eigenvector components (see Methods in main text). Left and right column show the case without and with coupling between the two sides respectively. The different rows correspond to different chiral symmetry breaking on-site energies on the left side of the chain.

D. Appearance of zeros in the SSH model

Supplementary Figure 11 shows how the zeros replace the poles of the Green's function as the dominating low-energy feature upon entering the Mott phase. At first zeros and poles are intermixed and only when U is large enough a clear structure of the zeros can be seen. Note that our analytic approach can only describe the very strong coupling limit (i.e. $U > 4$ in Supplementary Fig.11).

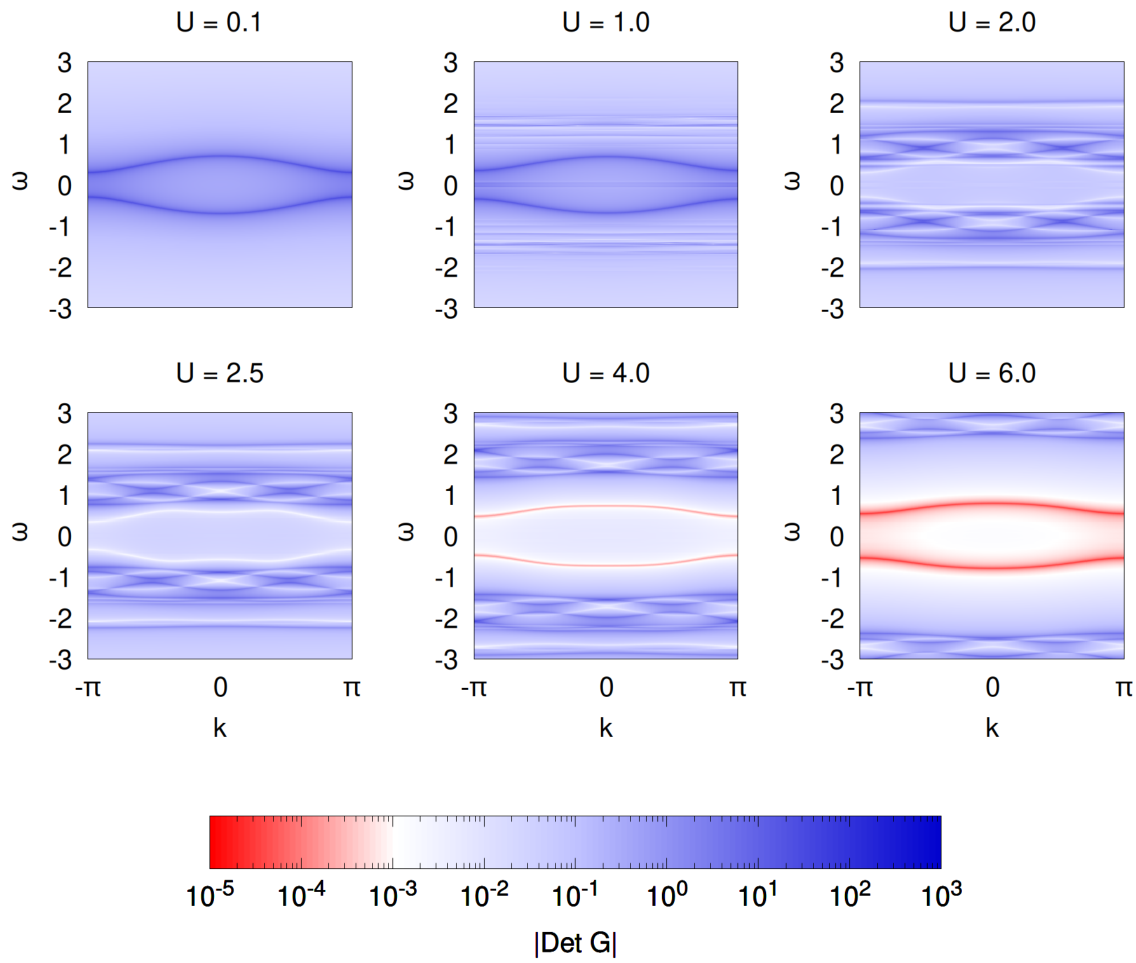

Supplementary Figure 11: Zero temperature CDMFT results for the SSH model for different interaction strengths U at half-filling. The SSH parameters are $v = 0.2$ and $w = 0.5$ and the cluster size is four sites.

E. Numerical estimation of higher order corrections

The results of Eq.(26) are applicable deep in the Mott phase. For smaller values of U, i.e. closer to the Mott transition, additional corrections become important. This is illustrated in Supplementary Fig.12 where CDMFT results for the SSH model are compared with a "truncated" version which is restricted to a few orders in a continued fraction of the form

$$\Sigma(k,\omega) = \frac{A}{\omega - B - \frac{C}{\omega - D - \frac{E}{\omega - F}}}. \tag{56}$$

Here A to F are coefficients which are determined by comparison with a moment expansion of the CDMFT self-energy. As can be seen in Supplementary Fig.12 the second order (taking all terms up to B into account), which corresponds to the analytic formula for the self-energy (Eq.(26)), becomes sufficient to describe the CDMFT results at large U. This also applies to the case of a Weyl semimetal (right-most plot in Supplementary Fig. 12, cf. Sec.Supplementary Note 3 K) where the zeros are gapless. Thus, although the derivation of the analytic formula is based on a high-frequency expansion in the limit of very strong interaction it is also capable of describing the low-energy features.
It is interesting to compare this new result with the known behavior of Lanczos calculations for Mott systems with a gap (see for instance Ref.[22], Chapter 7, pag. 25, Supplementary Fig. 4). There, one observes that the larger U is, the more floors one needs to obtain an accurate description of the spectrum, due to the more and more finely spaced excitations for large U and the wider and wider support of the spectrum. However, these lower-lying floors contribute only to improve the detailed structure of the outer parts of the Hubbard bands at large frequencies, while the main feature of the gap is determined by the first floors. This is totally in line with the conclusions of our new continued fraction expression for the self-energy in the Mott limit.

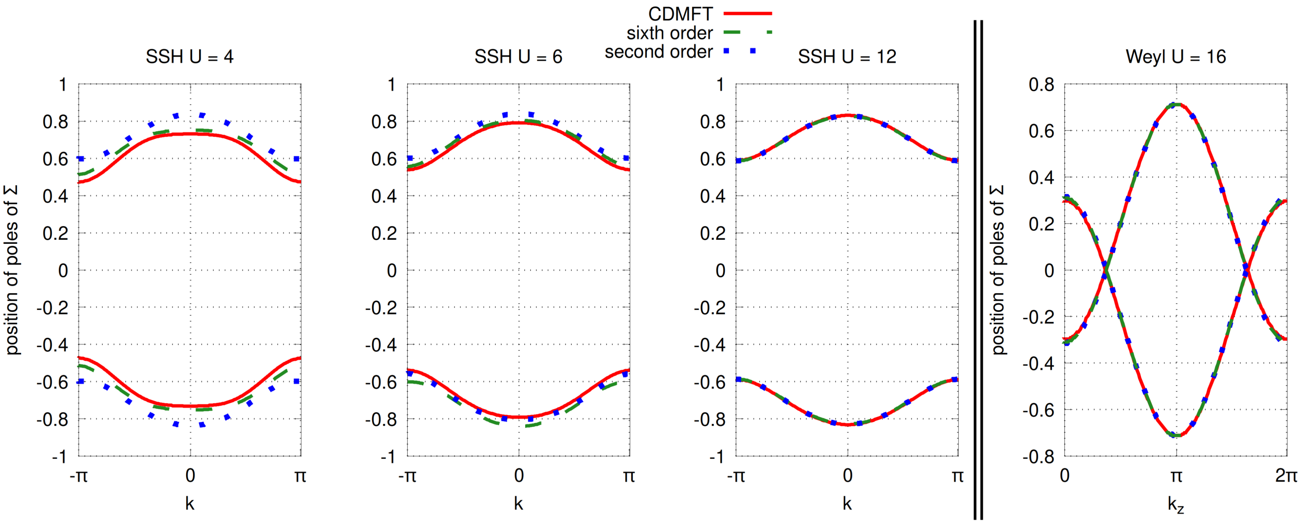

Supplementary Figure 12: Zero temperature CDMFT results for the poles of the self-energy for SSH models (hopping parameters: $v = 0.2$ and $w = 0.5$, CDMFT cluster size: 4 sites) with different values of interaction strength U. Dashed and dotted lines correspond to a truncation of the CDMFT results to a continued fraction using only the moments up to the indicated order. The sixth order corresponds to the full Eq.56, while the second order is taking only the terms up to B into account. The right-most plot shows the same analysis for a Weyl semimetal model (see Sec.Supplementary Note 3 K).

F. Tunneling through coupled region

In case of a finite coupled region between topological insulator and topological Mott insulator (see Supplementary Fig.13) a wave packet moving along an egde at the interface is not completely removed but continuous to propagate along the edge with reduced weight (see Supplementary Fig. 14).

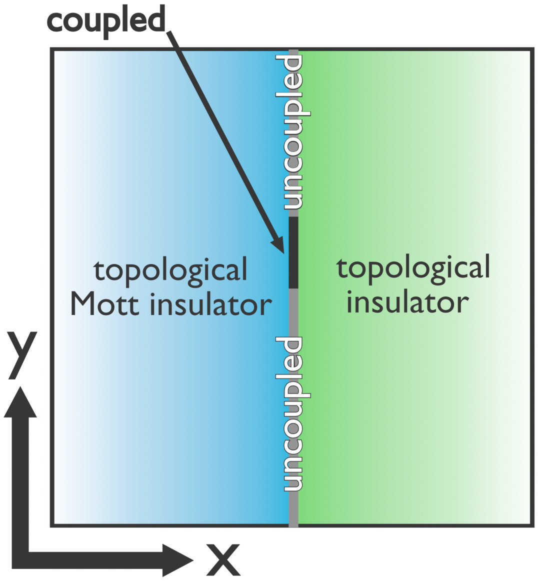

Supplementary Figure 13: Sketch of slab geometry with small coupled interface between topological insulator and topological Mott insulator.

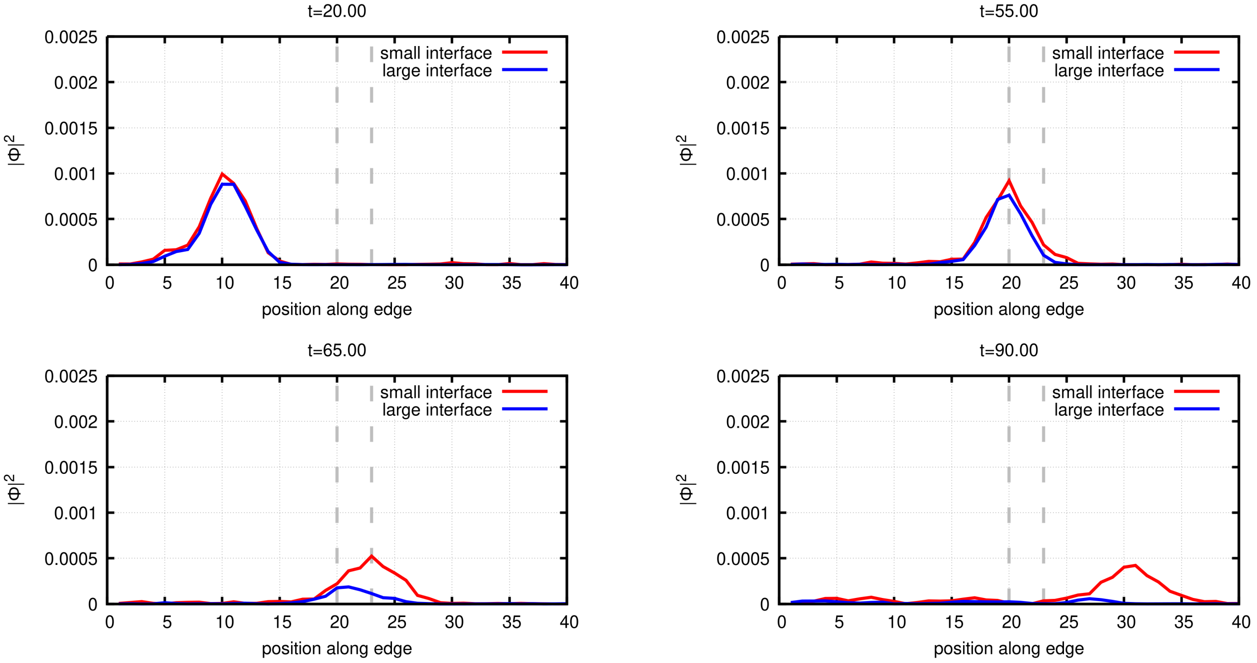

Supplementary Figure 14: Wave packet evolution along an interface between a topological Mott insulator and a topological insulator where the two sides are only coupled inside the region indicated with the dashed vertical lines (red). The blue line corresponds instead to the situation where the systems are coupled for all sites along the edge with an index larger then 20. See Supplementary movie 2.

G. Dependence of the pole-zero annihilation on the slope of the edge states

While the annihilation at the interface works best if the dispersion of edge pole and edge zero are the same, there is also a clearly visible effect when the slope differs. Supplementary Figure 15 shows the time evolution of wave packets where the slope of the zeros differs from the slope of the poles by factors α. These calculations are carried out in a slab geometry as described in Sec. Supplementary Note 2. For smaller α an increasing part of the wave packet survives but still the reduction of the total weight of the packet is clearly visible even for a large mismatch between the slopes. This gives some leeway for potential real material applications because no fine tuning of the edge slopes is required.

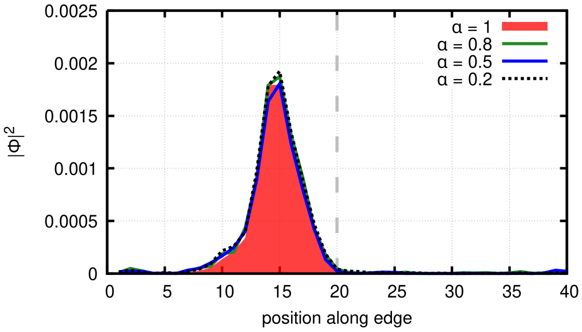

(a) $t = 35$

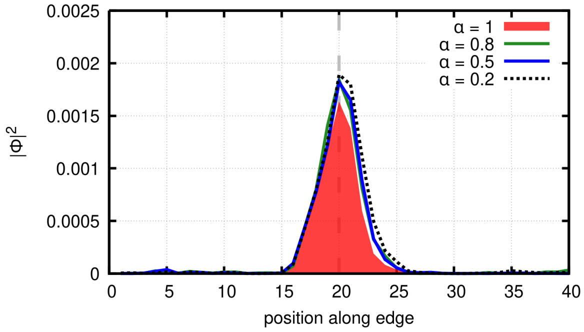

(b) $t = 54$

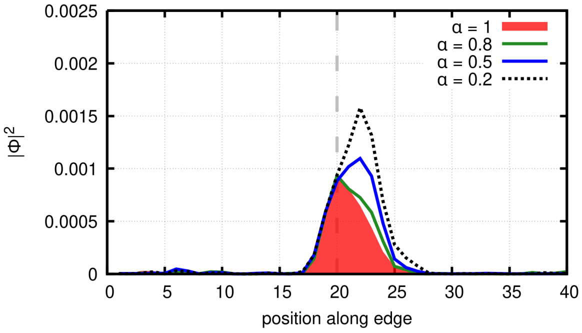

(c) $t = 60$

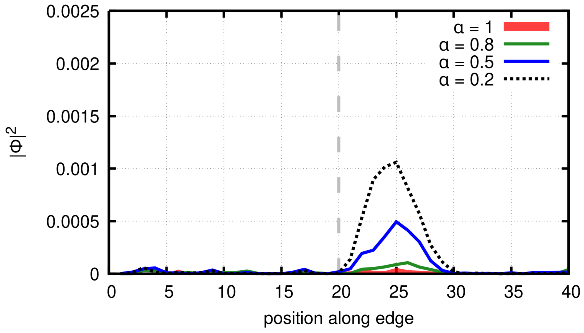

(d) $t = 70$

Supplementary Figure 15: Wave packet evolution along an interface between a topological Mott insulator and a topological insulator for different slopes α of the edge zero relative to the edge pole. See Supplementary movie 3.

H. Minimal model for edge annihilation

The annihilation at an interface can be investigated using the analytic formula for the self-energy (Eq.(26)) with a minimal non-interacting model given by

$$H = \begin{pmatrix} k & t \\ t & -k \end{pmatrix}, \tag{57}$$

describing edge pole and zero (called 'left' and 'right' respectively in order to keep the resemblance to an actual interface). For the 'right' site the dispersion has a minus which will be reverted for the corresponding zero. The coupling between pole and zero is given by t. The interaction is taken into account using Eq.(26) while setting all but the element corresponding to the 'right' site to zero. For the renormalization a flat factor α is used which creates a mismatch between the slopes of edge pole and edge state.

The annihilation process is illustrated in Supplementary Fig.16 using weighted eigenvector components and the spectral function. In the latter case the zeros are not directly visible, only the corresponding gap is recognizable. Supplementary Figure 17 shows the dependence of the annihilation on α. As long as $\alpha > 0$ the gapless state on the 'left' is removed upon coupling pole and zero. The size of the gap decreases when the value of α is reduced until at $\alpha = 0$ the 'left' state becomes gapless. The case $\alpha = 0$ corresponds to the case where the self-energy reduces to a single-site DMFT or atomic limit form, i.e. no momentum dependence.

Supplementary Figure 18 shows the spectral function and the weighted eigenvectors for different couplings t.

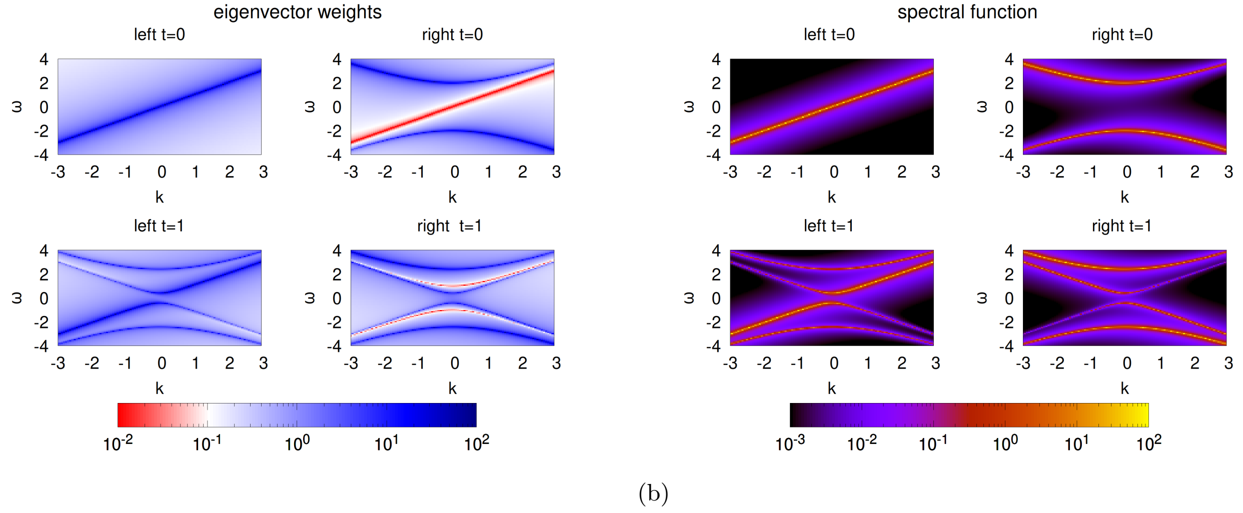

Supplementary Figure 16: Annihilation between zero and pole in a minimal model (Eq. (57) with $\alpha = 1$) describing edge pole and edge zero, coupled by t. In (a) weighted eigenvector components are shown and in (b) the spectral function. 'left' and 'right' refer to the two degrees of freedom the model has.

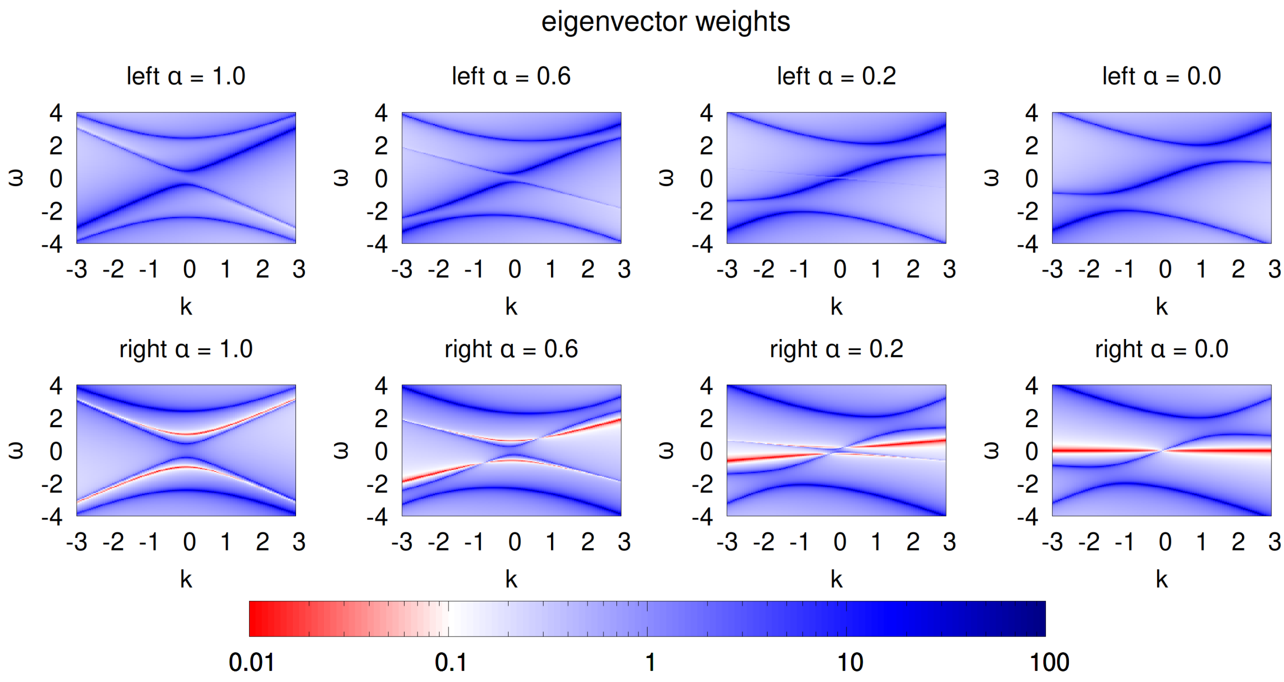

Supplementary Figure 17: Weighted eigenvector components showing the annihilation between zero and pole in a minimal model for different values of the slope α of the edge zero. 'left' and 'right' refer to the two degrees of freedom the model has.

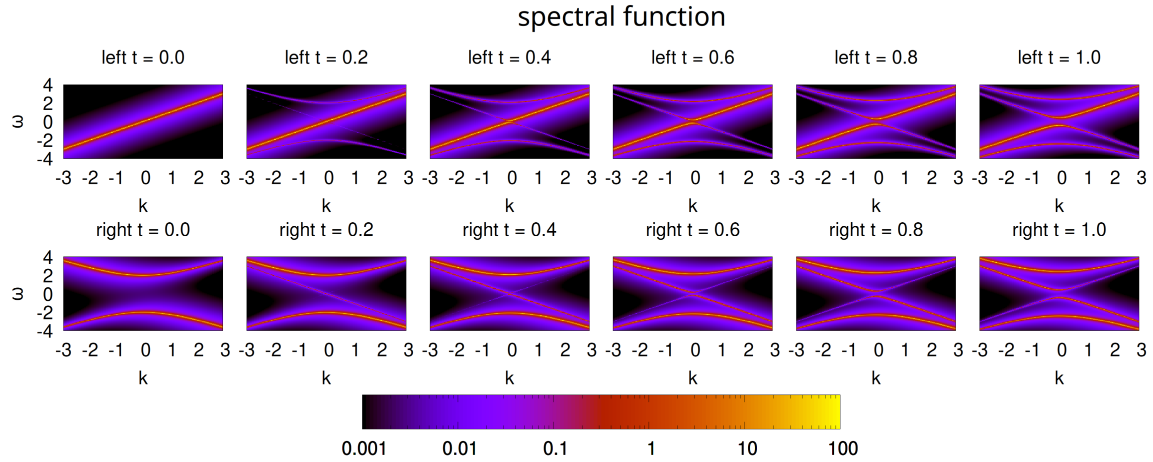

(a)

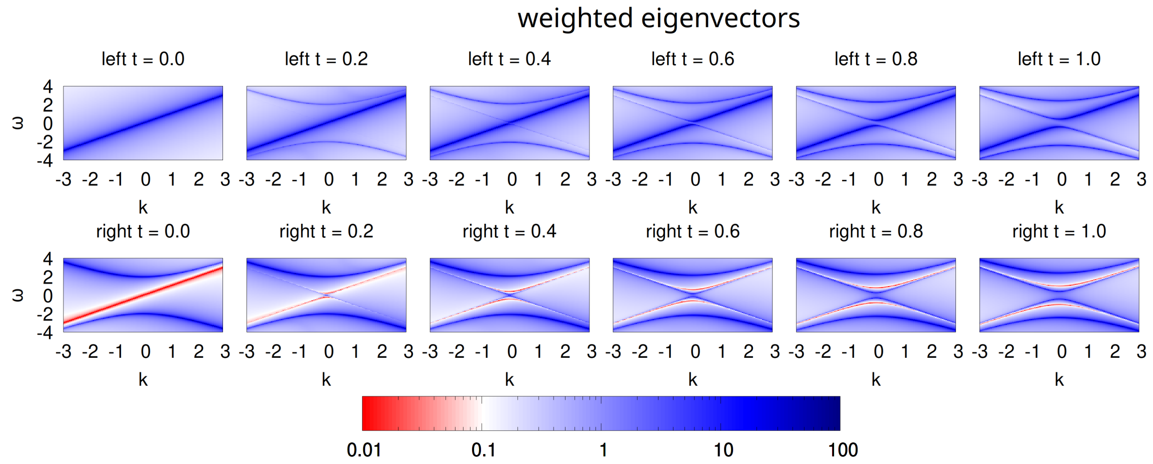

(b)

Supplementary Figure 18: Spectral function (a) and weighted eigenvector components (b) for the model Eq.(57) for different coupling strengths t. 'left' and 'right' refer to the two degrees of freedom the model has.

I. Orbital and spin character of the zeros

Analogously to the non-interacting band structure the zeros also retain an orbital/spin structure which is a consequence of the analytic self-energy formula Eq. (26). This is illustrated in Supplementary Fig. 19, showing the inverted (standard) gap in the zeros for a topological insulator (band insulator). Analogously, the zeros also have a spin character as shown in Supplementary Fig. 20. As a consequence of the plus sign in the denominator of Eq. 26, their spin character is interchanged between non-interacting and Mott system.

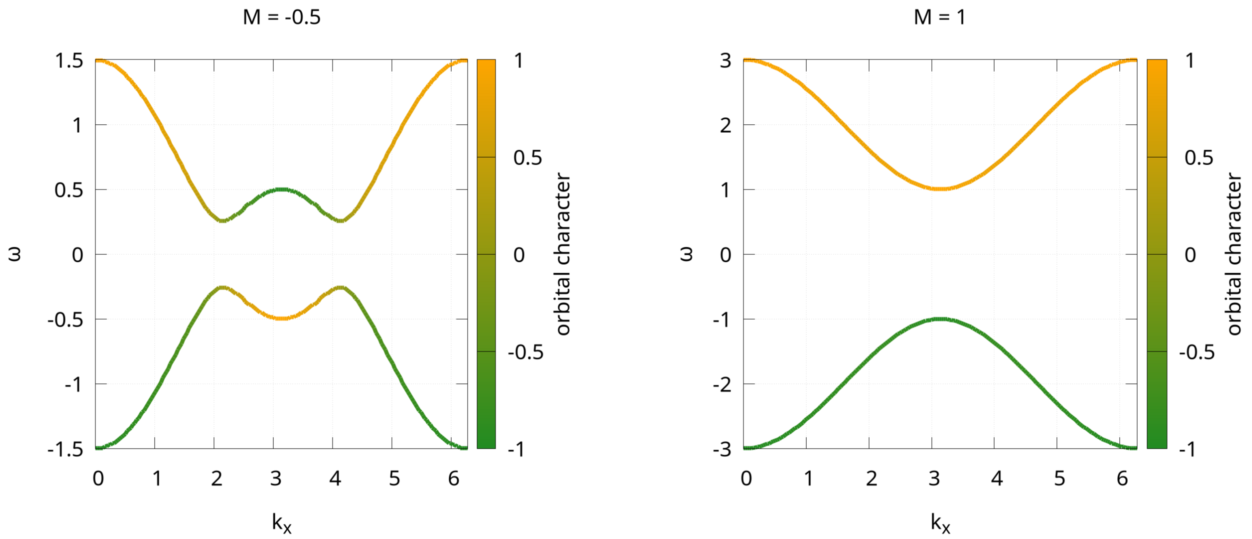

Supplementary Figure 19: Orbital character of the zeros obtained using the analytic formula Eq. (26) for a BHZ-model in the topological (left) and trivial (right) phase for $k_y = 0$, $t = 0.5$ and $\lambda = 0.3$ (see also Eq. (58) in section Supplementary Note 3 J).

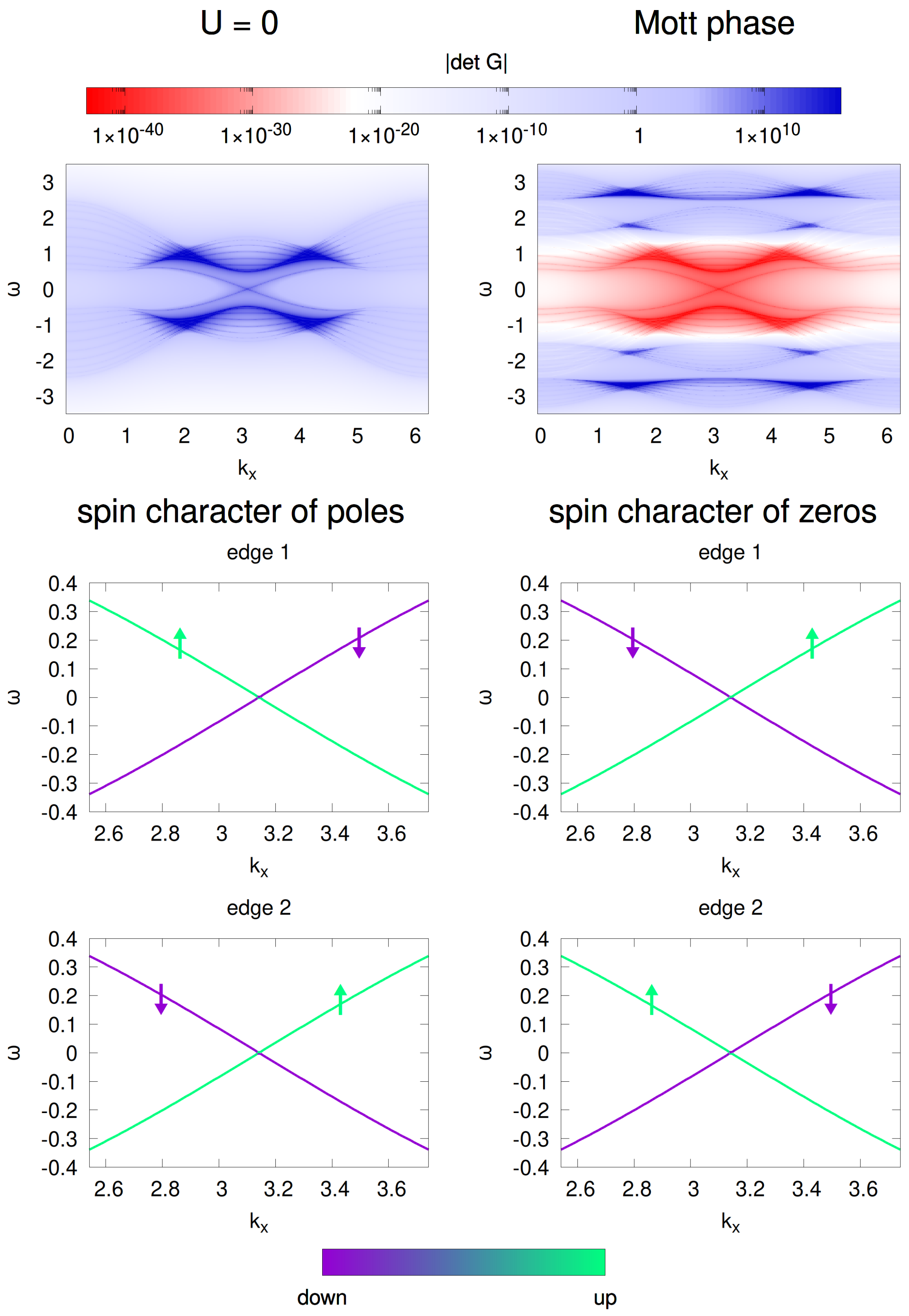

Supplementary Figure 20: BHZ model with finite size in y-direction and periodic boundary conditions in x-directions. The first row shows the poles and zeros for non-interacting and Mott Green's function. The interaction is included by using the analytic formula Eq.(26). The lower rows show the spin-momentum locking of the edge poles and zeros respectively for the two edges of the model. The second column shows data where M has the opposite sign compared to the non-interacting model in order to have the edge states/zeros at the same momenta.

J. Comparison of analytic formula and numerical results for a bulk BHZ model

We tested the analytic formula for a bulk BHZ model given by

$$H_0(k) = (M - 2t(\cos(k_x) + \cos(k_y))\tau_z + \lambda \sin(k_x)\tau_x + \lambda \sin(ky)\tau_y \quad (58)$$

where τ describes the orbital degree of freedom. In comparing the analytical dispersion with the CDMFT data, we have to be careful in including the effects the asymmetric cluster has on the results: computational memory constraints render a complete CDMFT simulation for a 4-site cluster (8 energy levels per spin, to be supplemented by at least as many bath levels) difficult to perform to convergence. We can however grasp the correctness of our analytical formula by considering a 2×1-site cluster, which breaks the C_2-symmetry of the square lattice. In this case, the self-energy will have nonlocal components in the x-direction, but not in the y direction, where the problem is effectively single-site. Hence, we expect the effective dispersion of the zeros to be comparable to an equation of type (58), once the terms depending on k_y have been completely neglected:

$$H_0^{2\times 1}(k) = (M - 2t\cos(k_x))\tau_z + \lambda \sin(k_x)\tau_x. \quad (59)$$

This will provide a gapped dispersion for $M = 0$, and a gap closing at $M = 1$ around the high-symmetry point Γ and around $M = -1$ around high-symmetry point X.
We fit the zeros of the CDMFT results with Eq. (59) using t and λ as fitting parameters, taking the signs due to the renormalization (Eq.(26)) into account. The crystal field-splitting M is not renormalized in the analytic self-energy formula and is therefore kept constant during the fit. As shown in Supplementary Fig. 21 the two approaches are in very good agreement. The renormalization of the parameters t and λ is about 0.5 which explains the shifting of the topological transition from $M = 1$ in the non-interacting case to $M \approx 0.5$. Note that for CDMFT we use a Kanamori-type interaction instead of the only-U case, which is used in the derivation of the analytic formula. In the only-U case the parameter λ is strongly suppressed, which is consistent with the high-frequency expansion, since the corresponding correlation values $\langle n_i n_j \rangle$ remain uncorrelated. This suggests that the analytic formula can also be applied to more complicated interactions as long as the correct behavior of the correlation values is taken into account.

Finally, we address the claim, mentioned in the main text, that in absence of perturbations that provoke the opening of gapless Dirac or Weyl points in the noninteracting Hamiltonian the zeros of the Green's function of the interacting system in the Mott phase remain gapless. We can verify this claim by making again use of the 2D BHZ model of eq. (58), which in the $M = 0$ case has a gapless dispersion at the X and Y high-symmetry points. As previously mentioned, a comprehensive analysis of the topological phase diagram for the BHZ model using a 2×2 cluster through CDMFT is hampered by the size of the impurity problem. Nevertheless, a converged result for selected M values is still within reach. In Supplementary Fig.22 we show the results after 10 DMFT steps for a 2×2 cluster for $M = 0$ as well as $M = 0.2$. As for the 2×1 cluster in the first part of this section we fit the zeros with the non-interacting dispersion using t and λ as fitting parameters. The resulting renormalization of the parameters is again given by a factor of about 0.5.

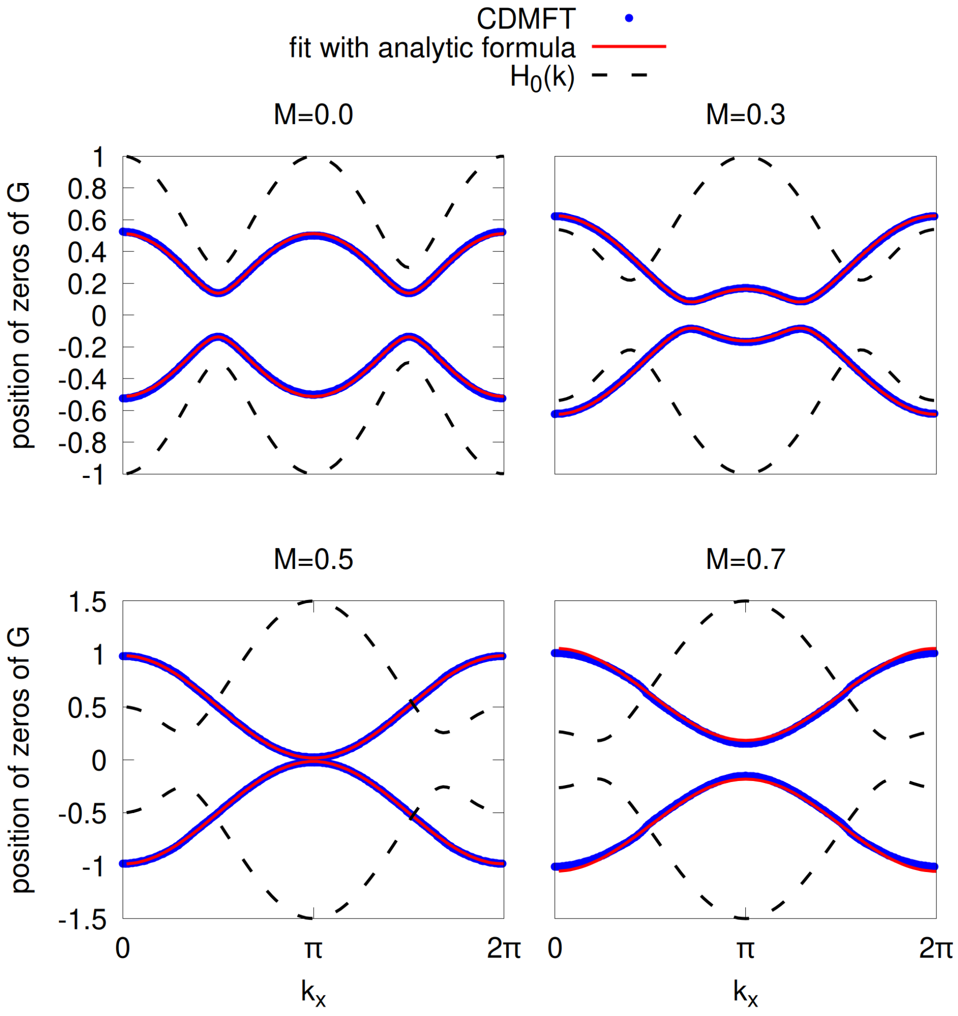

Supplementary Figure 21: Position of the zeros of G of a modified BHZ model (using a 2×1 cluster, see text) obtained using CDMFT ($U = 8$) and fits with the analytic formula for the self-energy. The dashed lines show the non-interacting band dispersion.

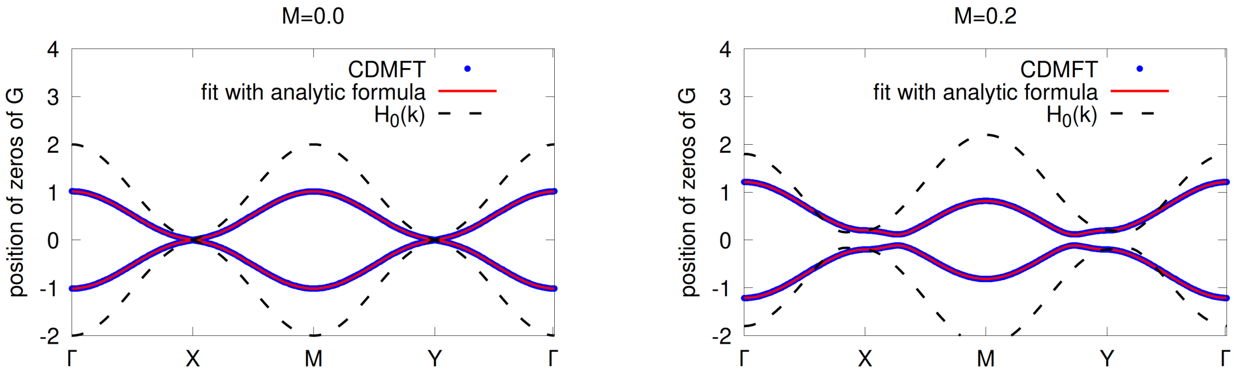

Supplementary Figure 22: Position of the zeros for a BHZ model obtained using CDMFT ($U = 8$) with a $2{\times}2$ cluster and fit with the analytic formula for the self-energy. Dashed lines show the non-interacting dispersion.

K. Comparison of analytic formula and numerical results for a bulk Weyl semimetal model

In order to check the predictions of our analytic approach for a Weyl semimetal we use a model adapted from Ref. [23]:

$$H_0 = (M - 2t(\cos(k_x) + \cos(k_y) + \cos(k_z))\tau_z + \lambda \sin(k_x)\tau_x + \lambda \sin(k_y)\tau_y. \tag{60}$$

Weyl points occur along k_z for $|M| <= 2$. For the CDMFT calculations we choose a 2-site cluster in z-direction. As a consequence we have to consider this geometry when comparing with the analytic formula (similar to the case for the BHZ model in Sec.Supplementary Note 3 J), leading to a Hamiltonian which determines the self-energy of the form

$$H_0^{1\times1\times2} = (M - 2t(\cos(k_z))\tau_z. \tag{61}$$

The result of a CDMFT calculation and a comparison with the analytic formula (using Eq. (61)) are shown in in Supplementary Fig. 23. The evolution of the zeros upon changing the mass parameter M is displayed in Supplementary Fig. 24, demonstrating the applicability of the analytic approach to this system. For the fit the value of M is kept fixed and t is used as a fitting parameter which gets renormalized by a factor of roughly 0.5 (also taking the minus sign according to Eq.(26) into account).
These results confirm our claim that the zeros contain the protected structures of the non-interacting system, in this case the linear Weyl crossing.

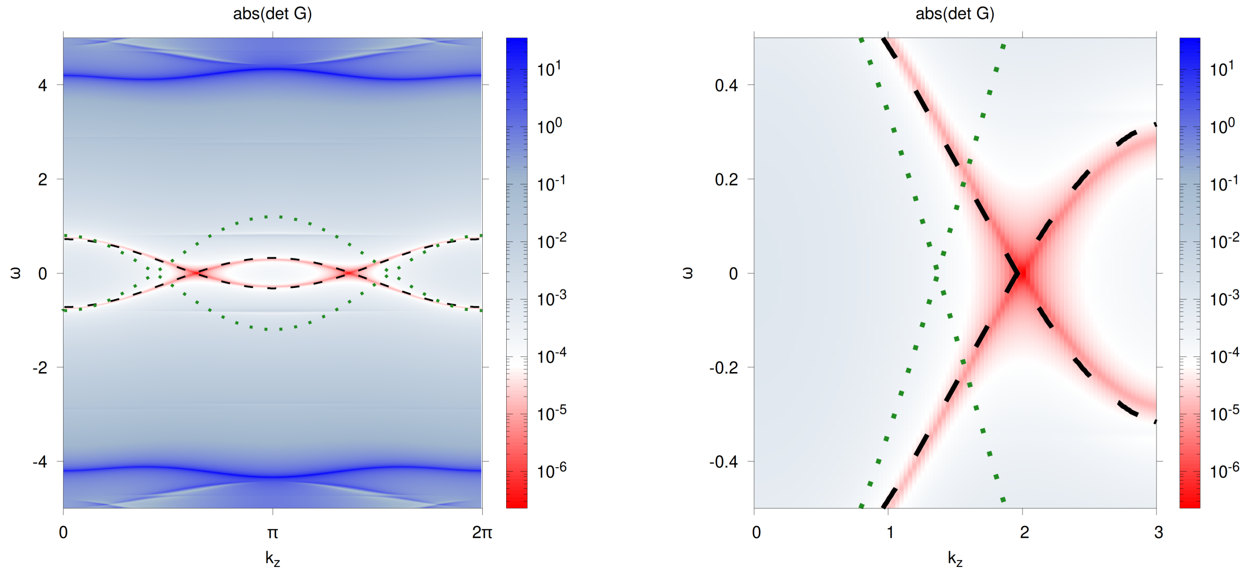

Supplementary Figure 23: CDMFT results for the determinant of the Green's function for a Weyl semimetal model for $U = 8$. Dashed black lines are fits with the analytic formula for the self-energy and dashed green lines indicate the non-interacting band structure (Eq.(61)). The parameters are $M = 0.2$, $t = 0.5$ and $\lambda = 0.3$. The plot on the right hand side is a zoom on a Weyl point.

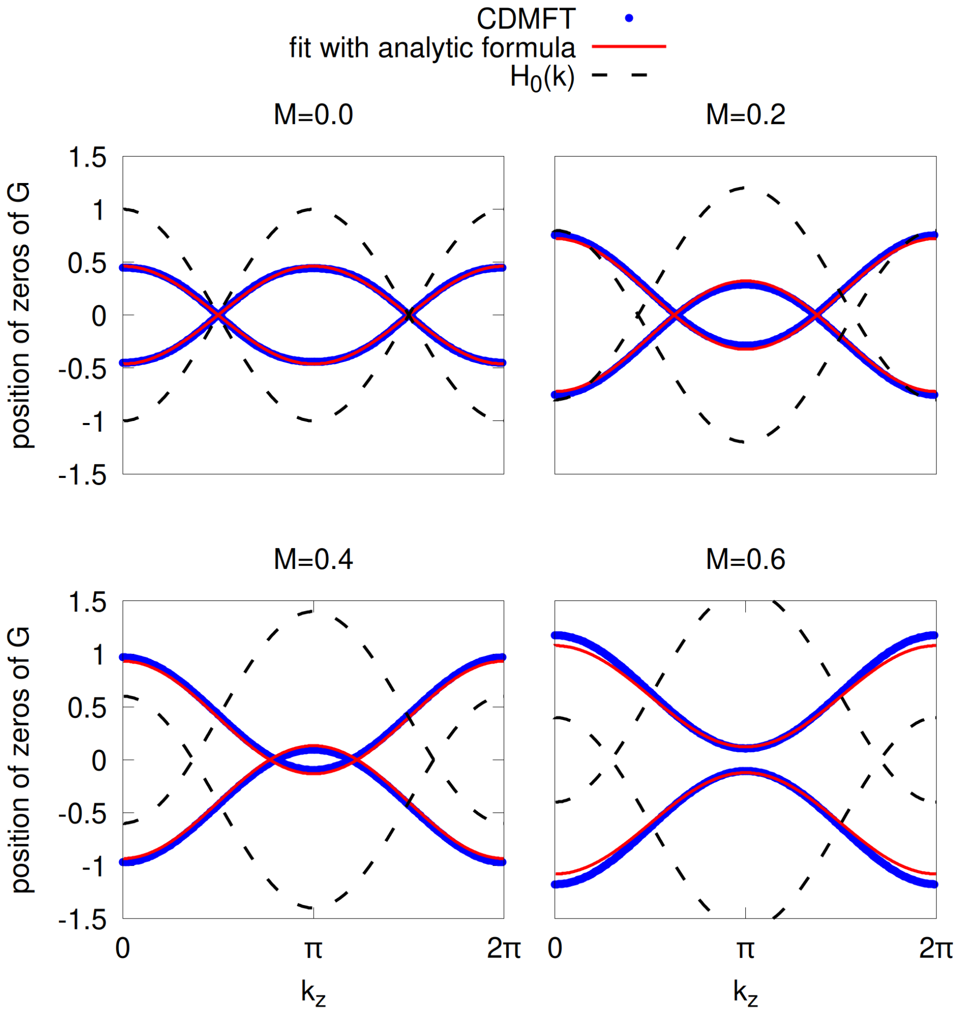

Supplementary Figure 24: Position of the zeros of G of a Weyl semimetal model (using a 2-site cluster in z-direction, see text) obtained using CDMFT ($U = 8$) and fits with the analytic formula for the self-energy (using Eq. (26) with Eq.(61). The parameters of the non-interacting system are $t = 0.5$ and $\lambda = 0.3$. Dashed lines show the corresponding band dispersion.

L. Spin gap in the SSH model

As discussed in the main text, for large interaction the topological behavior of the SSH model results in gapless edge zeros. The presence of these zeros can be connected to gapless spin excitations as can be seen in Supplementary Fig.25(a) (see also Ref.[24–26]) which shows the spin gap obtained by solving a finite Heisenberg chain with staggered exchange interaction using ED. The Hamiltonian is given by

$$H = \sum_i J_i \vec{S}_i \cdot \vec{S}_{i+1} \tag{62}$$

where $\vec{S}_i$ is the vector of spin-operators at site i and the exchange interaction is given by

$$J_i = \begin{cases} J_0 - \delta J & \text{for } i \text{ even} \\ J_0 + \delta J & \text{for } i \text{ odd} \end{cases} \tag{63}$$

As long as the chain is in the trivial phase ($\delta J/J_0 > 0$), the spin-gap is finite. Instead, in the topological non-trivial phase ($\delta J/J_0 < 0$), the spin-gap is always zero which can be understood by the presence of free spins at the edge. In the case of periodic boundary conditions the spingap closes only at the topological phase transition, i.e. where the gap in the bulk zeros closes. We have confirmed these results by computing the spin gap for the Hubbard model, i.e. without restricting the Hilbert space to single occupied sites (see Supplementary Fig.25(b)).

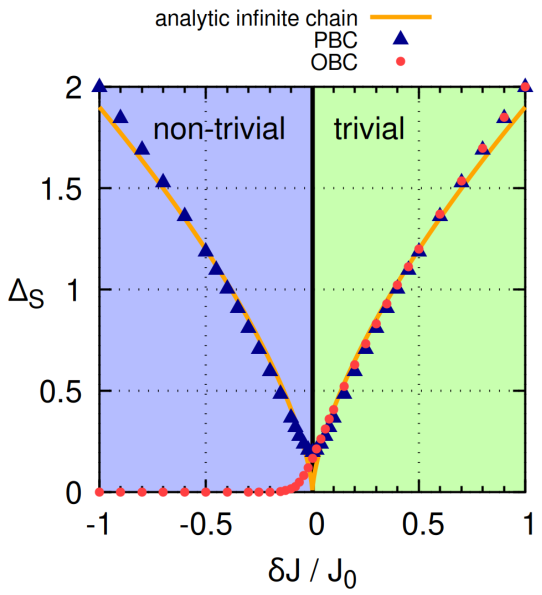

(a) Heisenberg model

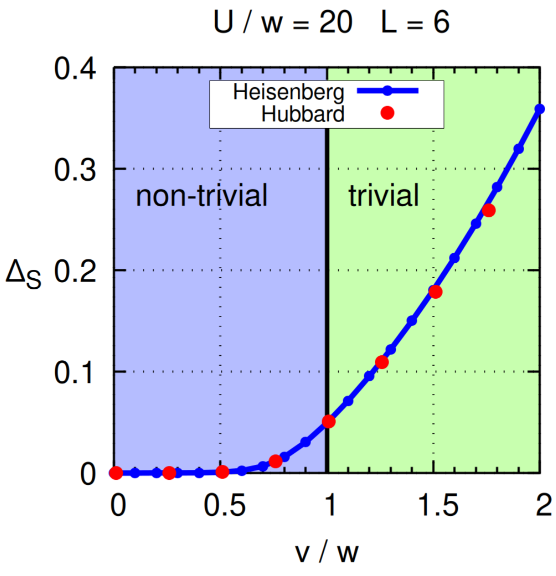

(b) Heisenberg model vs Hubbard model

Supplementary Figure 25: (a) ED results for the spin-gap of a SSH-Heisenberg-model for a 26-site chain. Red points correspond to open boundary conditions and blue triangles to periodic boundary conditions. The difference between the two staggered hoppings is given by δJ. The orange line indicates the analytic result for an infinite chain [27]. See Supplementary Fig.26 for an analysis of the dependence of the results on the length of the chain. (b) Comparison between the ED results obtained for the Hubbard model and the Heisenberg model for a $L = 6$ chain with $U/w = 20$. For the Heisenberg model the hopping parameters are translated to exchange interactions as v^2/U and w^2/U.

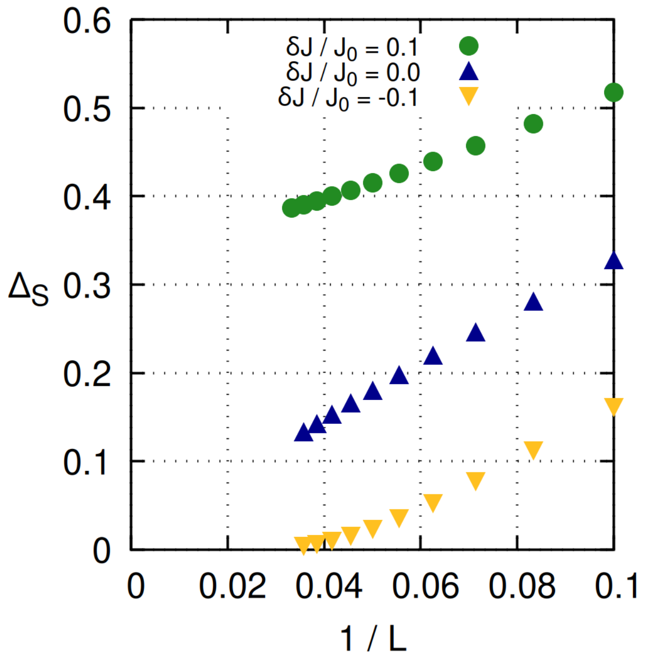

Supplementary Figure 26: Scaling behavior of the spin-gap as a function of chain length L for the Heisenberg model solved with ED. Since here we have open boundary conditions, the negative-δJ curve scales to zero, as opposed to the positive δJ. The extrapolation of the $\delta J = 0$ curve to zero for large L is clear from the data.

[1] W. Nolting, Methode der Spektralmomente für das Hubbard-Modell eines schmalen S-Bandes, Zeitschrift für Physik A Hadrons and nuclei **255**, 25 (1972).
[2] A. B. Harris and R. V. Lange, Single-Particle Excitations in Narrow Energy Bands, Physical Review **157**, 295 (1967).
[3] L. M. Roth, Electron Correlation in Narrow Energy Bands. I. The Two-Pole Approximation in a Narrow S Band, Physical Review **184**, 451 (1969).
[4] S. Onoda and M. Imada, Mott transitions in the two-dimensional half-filled Hubbard model: Correlator projection method with projective dynamical mean-field approximation, Physical Review B **67**, 161102 (2003).
[5] A. Avella and F. Mancini, The Composite Operator Method (COM), in *Strongly Correlated Systems*, Vol. 171, edited by A. Avella and F. Mancini (Springer Berlin Heidelberg, Berlin, Heidelberg, 2012) pp. 103–141, series Title: Springer Series in Solid-State Sciences.
[6] A. Avella, The Hubbard model beyond the two-pole approximation: a composite operator method study, The European Physical Journal B **87**, 45 (2014).
[7] G. Emanuel, *Continuous-Time Quantum Monte Carlo Algorithms for Fermions*, Ph.D. thesis, ETH Zurich (2008).
[8] E. Gull, A. J. Millis, A. I. Lichtenstein, A. N. Rubtsov, M. Troyer, and P. Werner, Continuous-time Monte Carlo methods for quantum impurity models, Reviews of Modern Physics **83**, 349 (2011).
[9] K. Haule, Quantum Monte Carlo impurity solver for cluster dynamical mean-field theory and electronic structure calculations with adjustable cluster base, Physical Review B **75**, 155113 (2007).
[10] S. Pairault, D. Sénéchal, and A.-M. Tremblay, Strong-coupling perturbation theory of the Hubbard model, The European Physical Journal B **16**, 85 (2000).
[11] R. Strack and D. Vollhardt, Dynamics of a hole in the t - J model with local disorder: Exact results for high dimensions, Physical Review B **46**, 13852 (1992).
[12] G. Sangiovanni, A. Toschi, E. Koch, K. Held, M. Capone, C. Castellani, O. Gunnarsson, S.-K. Mo, J. W. Allen, H.-D. Kim, A. Sekiyama, A. Yamasaki, S. Suga, and P. Metcalf, Static versus dynamical mean-field theory of Mott antiferromagnets, Physical Review B **73**, 205121 (2006).
[13] While our paper was under review, and this supplement was not yet made publically available, Blason and Fabrizio posted independently a preprint with substantial overlap [28].
[14] Z. Wang, X.-L. Qi, and S.-C. Zhang, Topological Order Parameters for Interacting Topological Insulators, Physical Review Letters **105**, 256803 (2010).
[15] Z. Wang and S.-C. Zhang, Simplified Topological Invariants for Interacting Insulators, Physical Review X **2**, 031008 (2012).
[16] B. Ware, I. Kimchi, S. A. Parameswaran, and B. Bauer, Topological crystalline Bose insulator in two dimensions via entanglement spectrum, Physical Review B **92**, 195105 (2015).
[17] T. Fukui, Y. Hatsugai, and H. Suzuki, Chern Numbers in Discretized Brillouin Zone: Efficient Method of Computing (Spin) Hall Conductances, Journal of the Physical Society of Japan **74**, 1674 (2005).
[18] J. M. Tomczak, Spectral and Optical Properties of Correlated Materials (2007).
[19] L. Fu and C. L. Kane, Topological insulators with inversion symmetry, Physical Review B **76**, 045302 (2007).
[20] T. Yoshida, S. Fujimoto, and N. Kawakami, Correlation effects on a topological insulator at finite temperatures, Physical Review B **85**, 125113 (2012).
[21] Y. Ando, Topological Insulator Materials, Journal of the Physical Society of Japan **82**, 102001 (2013).
[22] E. Pavarini, E. Koch, and S. Zhang, eds., *Many-Body Methods for Real Materials*, Schriften des Forschungszentrums Jülich. Modeling and Simulation, Vol. 9 (Forschungszentrum Jülich GmbH Zentralbibliothek, Verlag, Jülich, 2019) backup Publisher: Autumn School on Correlated Electrons, Jülich (Germany), 16 Sep 2019 - 20 Sep 2019.
[23] N. Armitage, E. Mele, and A. Vishwanath, Weyl and Dirac semimetals in three-dimensional solids, Reviews of Modern Physics **90**, 015001 (2018).
[24] T. Yoshida, R. Peters, S. Fujimoto, and N. Kawakami, Characterization of a Topological Mott Insulator in One Dimension, Physical Review Letters **112**, 196404 (2014).
[25] M. P. Estarellas, I. D'Amico, and T. P. Spiller, Topologically protected localised states in spin chains, Scientific Reports **7**, 42904 (2017).
[26] N. H. Le, A. J. Fisher, N. J. Curson, and E. Ginossar, Topological phases of a dimerized Fermi–Hubbard model for semiconductor nano-lattices, npj Quantum Information **6**, 24 (2020).
[27] T. Giamarchi, *Quantum Physics in One Dimension* (Oxford University Press, 2003).
[28] A. Blason and M. Fabrizio, Unified role of Green's function poles and zeros in topological insulators (2023), arXiv:2304.08180 [cond-mat].
