# Supplementary figures and images for: Mott insulators with boundary zeros
Source: Nat Commun. 2023 Nov 20;14:7531. doi: 10.1038/s41467-023-42773-7 (PMC10662449; doi:10.1038/s41467-023-42773-7)

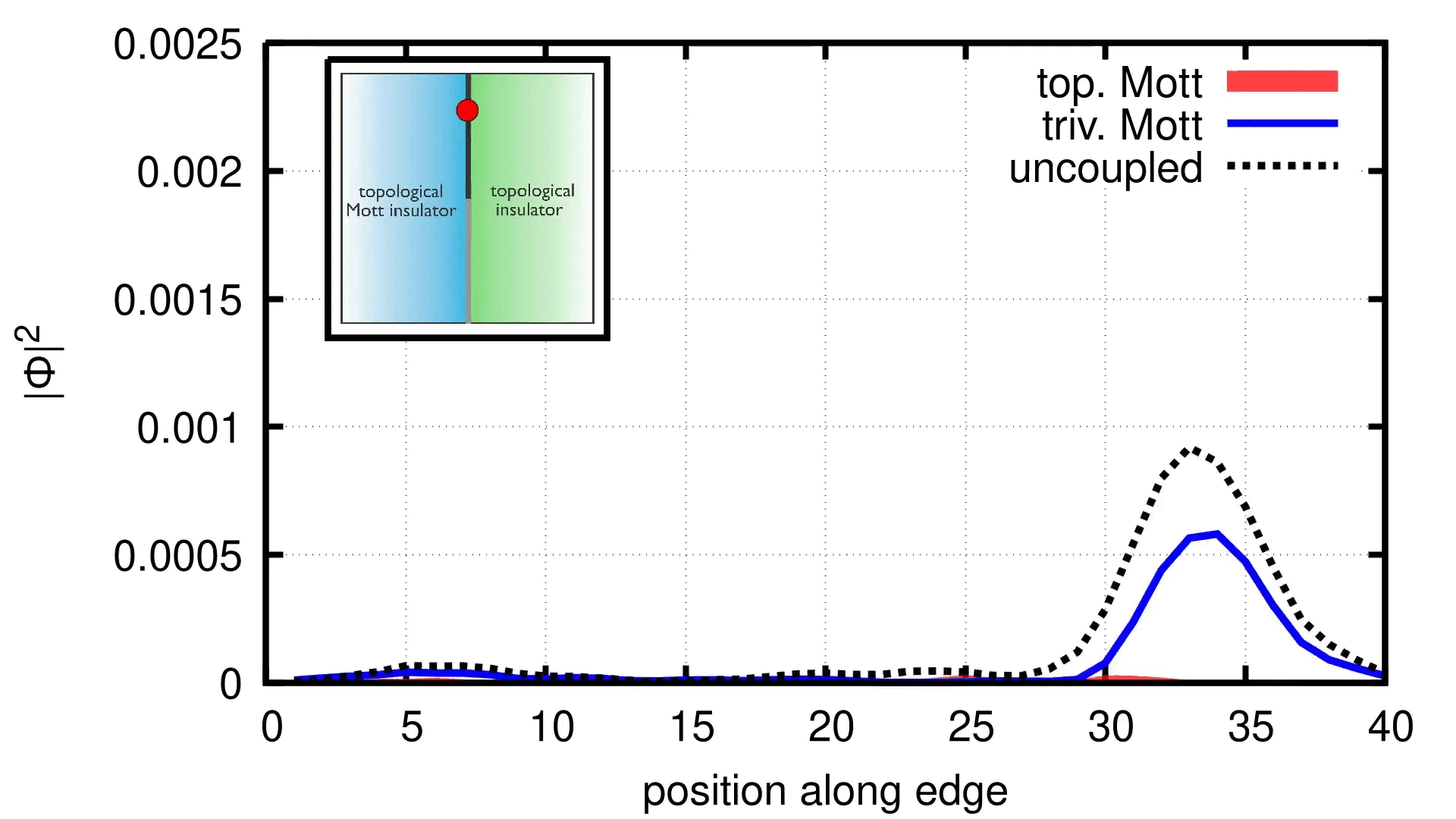

Supplement: Supplementary file 3 — Supplementary Movie 1 [file 41467_2023_42773_MOESM3_ESM.gif]

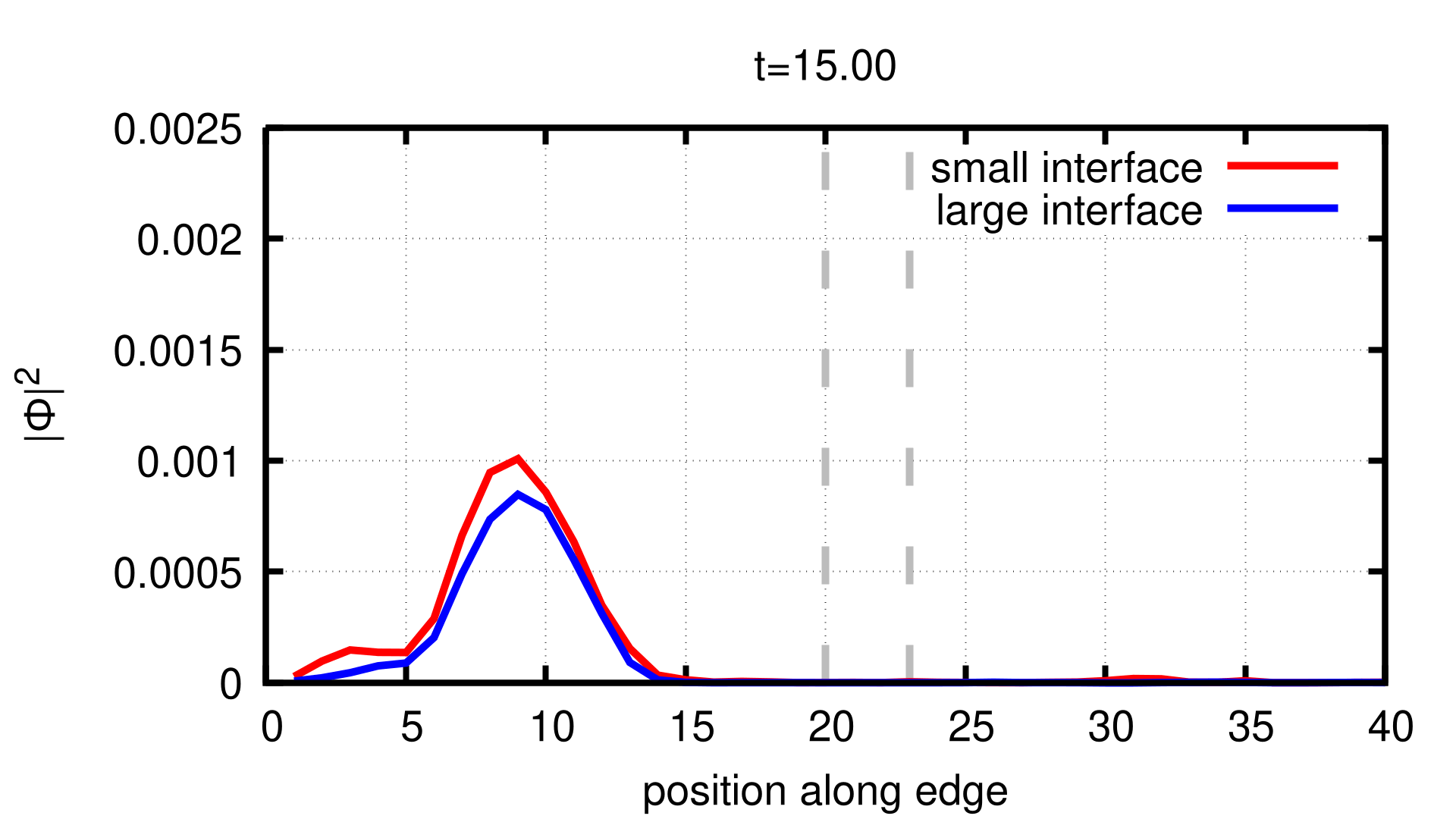

Supplement: Supplementary file 4 — Supplementary Movie 2 [file 41467_2023_42773_MOESM4_ESM.gif]

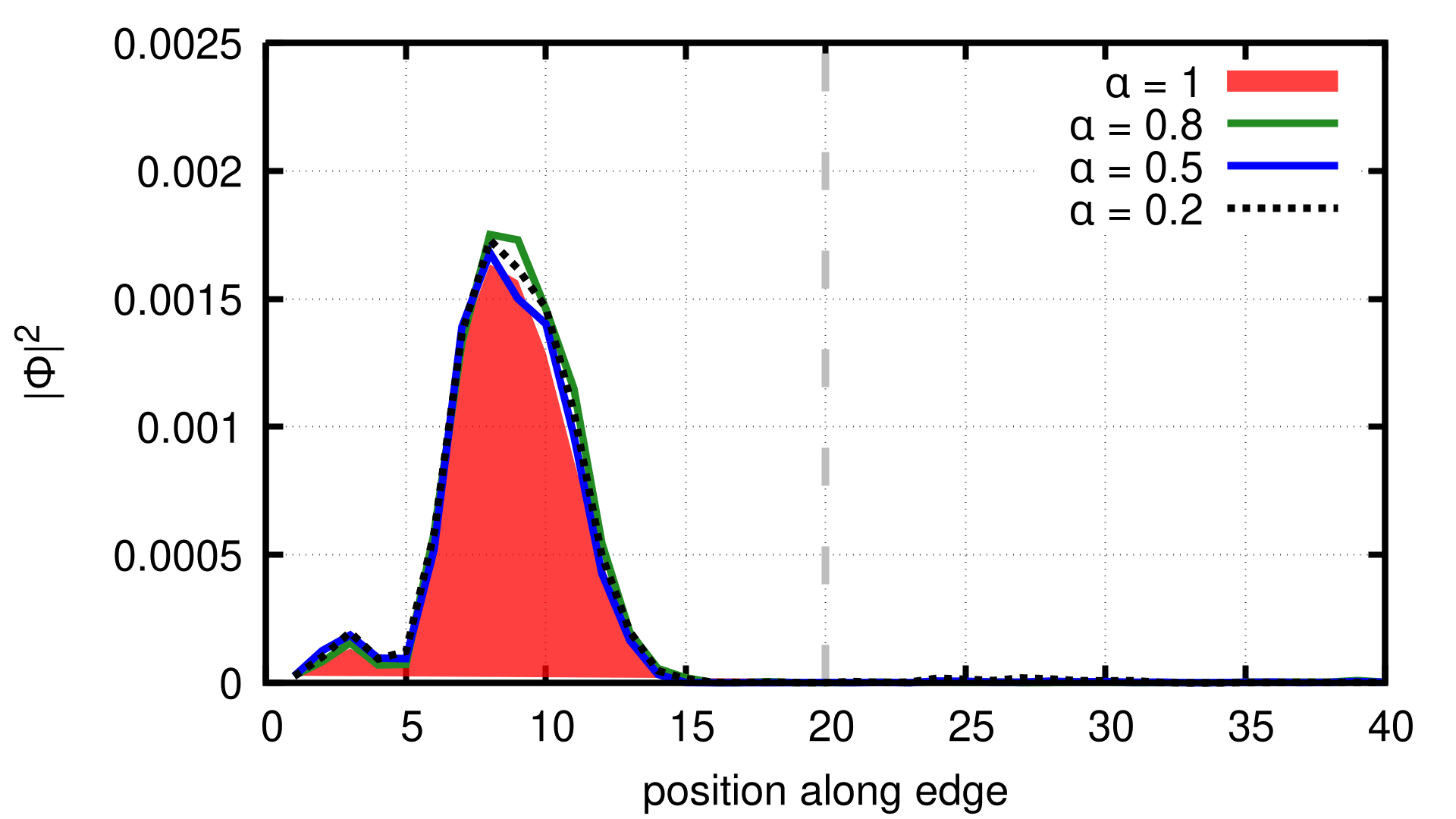

Supplement: Supplementary file 5 — Supplementary Movie 3 [file 41467_2023_42773_MOESM5_ESM.gif]
